# Genome-wide Identification and Structural, Functional and Evolutionary Analysis of WRKY Components of Mulberry

**DOI:** 10.1038/srep30794

**Published:** 2016-08-01

**Authors:** Vinay Kumar Baranwal, Nisha Negi, Paramjit Khurana

**Affiliations:** 1 Department of Plant Molecular Biology, University of Delhi South Campus, Benito Juarez Road, New Delhi, 110021, India

## Abstract

Mulberry is known to be sensitive to several biotic and abiotic stresses, which in turn have a direct impact on the yield of silk, because it is the sole food source for the silk worm. WRKYs are a family of transcription factors, which play an important role in combating various biotic and abiotic stresses. In this study, we identified 54 genes with conserved WRKY motifs in the *Morus notabilis* genome. Motif searches coupled with a phylogenetic analysis revealed seven sub-groups as well as the absence of members of Group Ib in mulberry. Analyses of the 2K upstream region in addition to a gene ontology terms enrichment analysis revealed putative functions of mulberry WRKYs under biotic and abiotic stresses. An RNA-seq-based analysis showed that several of the identified WRKYs have shown preferential expression in the leaf, bark, root, male flower, and winter bud of *M. notabilis*. Finally, expression analysis by qPCR under different stress and hormone treatments revealed genotype-specific responses. Taken together, our results briefs about the genome-wide identification of WRKYs as well as their differential response to stresses and hormones. Importantly, these data can also be utilized to identify potential molecular targets for conferring tolerance to various stresses in mulberry.

Plants, being sessile, have developed different molecular mechanisms to avoid or cope with different types of biotic and abiotic stresses. Numerous transcription factors, which confer tolerance to different types of stresses, have been identified and characterized. The WRKY family of transcription factors is one such example, and their genes exhibit a very diverse range of expression. WRKY genes have been found to regulate physiological developmental in plants. WRKYs regulate numerous downstream genes by binding to W-box sequences present in the promoter region. Structurally, WRKYs typically contain one or two WRKY domains approximately 60 amino acids in length along with C_2_H_2_ or C_2_HC zinc-finger motifs. Based on the distribution of these conserved domains, WRKY genes have been classified in to three groups^1^. Moreover, based on the type and distribution of zinc finger motifs into different clades, WRKYs are divided into sub-groups. Group Ia WRKYs contain two conserved WRKY domains along with two C_2_H_2_ conserved Zn fingers^2^. Group Ib WRKYs contain two WRKY domains and two C_2_HC motifs^2^. Groups II and III have been shown to possess one conserved WRKY domain each, and a Zn finger motif of C_2_H_2_ and C_2_HC type, respectively^2^. Group IV WRKYs are divided into two sub-groups, based on the presence or absence of an imperfect zinc finger motif. The evolution of WRKY genes in multiple organisms has been well-studied and phylogenetic analysis has shown that these genes evolved through multiple rounds of duplication, leading to the evolution of new groups. For example, Group III members are absent in *Physcomitrella patens*^3^. WRKY genes have not been reported in archea, eubacteria, eukaryotic fungi, or animals. However, these genes are present in the protists (*Giardia lamblia*) and slime mould (*Dictyostelium discoideum*)^3^. From an evolutionary standpoint, Group I WRKY genes are considered the most ancient, because they are well-represented in early organisms, such as protists. The functional roles of several WRKY genes have been elucidated in plants subjected to biotic and abiotic stress. Under biotic stress, WRKYs play a major role in regulating senescence^4^. Their roles in herbivory^5^, aphid resistance^6^, and other biotic resistance mechanisms are also well known^7^. Alternatively, the role of WRKYs under abiotic stress, including heat^8^,^9^, drought^10^,^11^, and cold stress^12^, has also been established in a wide variety of organisms. From a developmental perspective, WRKYs have been shown to be involved in lateral root^13^ and trichome development^14^ in addition to bud and floral differentiation 15. The WRKY genes expression also plays an important role in secondary metabolism^16^,^17^. Furthermore, the physiological responses modulated by these WRKYs include auxin-13,18, cytokinin-19, jasmonic acid-, and salicylic acid-mediated responses^20^,^21^,^22^. Thus, these transcription factors have a wide range of activities, functioning chiefly as key regulators of various mechanisms in plants.

Mulberry is an important tree species, on which almost the entire silk industry depends. However, mulberry does have other, very diverse uses, such as in timber, dye, wood, and pulp industries. The recent publication of the draft genome sequence of mulberry^23^ has provided researchers with an opportunity to determine the role of WRKY transcription factors in this economically important species. The availability of RNA-seq data has allowed for analysis of the WRKY expression profile at various developmental stages as well as in contrasting varieties of mulberry. In this study, we characterized the WRKY gene family in mulberry, studied its expression patterns and conserved domains, and performed gene ontology (GO) enrichment analysis and phylogenetic analysis to determine its structural, functional, and evolutionary aspects. We chose three species, from varied geographical backgrounds, for differential expression analysis. The first was *Morus notabilis* (MN), which grows in the wild and is a native of the Sichuan province in China. It harbors numerous genes related to biotic and abiotic stress^24^,^25^. The second, *M. laevigata* (ML), is distributed throughout India and the third, *M. serrata* (MS) is restricted to the Himalayan foothills. Both ML and MS are resistant to *Phyllactinia corylea*, an Erysiphaceae member causing powdery mildew of mulberry. Compared to MS, ML is a hardy tree species and is widely used for timber and forage^26^. We performed a comparative expression profile of WRKY genes in the leaf tissues of these three species. Furthermore, we conducted real-time PCR based on the expression of select WRKYs, which were found to be differentially expressed under different stress and hormonal treatments.

## Methods

### Identification of WRKY gene family members

WRKY transcription factors were identified using two approaches, hmm and BLAST homology search. The mulberry proteome was downloaded from MorusDB^27^. A hundred and two predicted rice WRKY protein sequences were downloaded from the RGAP Database (http://rice.plantbiology.msu.edu/) and 74 *Arabidopsis* WRKY protein sequences were been downloaded from the TAIR Database (https://www.arabidopsis.org/tools/bulk/sequences/index.jsp). An hmm profile of WRKY domain (accession number PF03106.10) was used to query the predicted mulberry proteome using hmmscan (hmm suit version 3.12b2). Furthermore, blastp searches with known rice and *Arabidopsis* WRKY sequences were carried out. The results obtained were combined and searched for the presence of WRKY domains using NCBI-CDD (http://www.ncbi.nlm.nih.gov/Structure/cdd/cdd.shtml)^28^ and ScanProsite (http://prosite.expasy.org/scanprosite/)^29^. The identified WRKY domains were fetched using a custom-written perl script, employing samtools version 1.1^30^, and were used to create a custom hmm profile, using hmmbuild of the hmm suite. This custom profile was used to query the proteome again. These steps were repeated until no new members could be identified.

### WRKY sequence alignment and phylogenetic analysis

For phylogenetic analysis, the identified mulberry protein sequences were aligned using Clustal X version 2.1. Based on this alignment, a bootstrapped NJ tree was constructed using MEGA version 6.06. To assess the phylogenetic relationships amongst the members of the mulberry WRKY gene family, a phylogenetic tree was prepared based on the alignment of only mulberry proteins. All WRKY transcription factors were classified into subgroups, based on their alignment and domain distribution. The predicted WRKY domains were fetched using a custom-written perl script and was aligned in Clustal X 2.1 to identify the WRKY motif and its variants in mulberry.

### Structural analysis of genes and putative promoters

Gene and mRNA sequences of mulberry WRKYs were downloaded from MorusDB^27^. Gene Structure Display Server ver 2.0^31^ was used to display the intron and exon junction as well as the arrangements of these genes. Molecular mass and isoelectric focusing point estimations were performed using custom-written perl scripts, employing bioperl modules^32^. Other motifs present in these sequences were identified using the MEME suite (http://meme-suite.org/)^33^ and hmmscan (hmm suite version 3.12b2). Search criteria for the identification of motifs using MEME included 15 motifs, with an optimum motif width between 8 and 50 residues, with any number of repetitions. The predicted conserved motifs were annotated using an InterProScan database search (http://www.ebi.ac.uk/Tools/pfa/iprscan/). GO terms for these WRKY genes were fetched and a hypergeometric distribution test analysis was conducted using the Bingo plugin^34^ and the GO networks were displayed using Cytoscape version 3.02^35^. Two kb upstream sequences were fetched from the assembled scaffolds from MorusDB^27^. These 2 kb upstream sequences were further searched for the *cis*-acting motifs, which were downloaded from PLACE database^36^. The subsequently identified motifs were input in the ‘wordcloud’ package of R version 2.5. Furthermore, important motifs that were verified in other systems were shown using a diagram generated using a custom-written gff file.

### Differential expression analyses

RNA-seq data of ML and MS were retrieved from the NCBI Sequence Read Archive (SRP068061 and SPR067869, respectively), which was previously sequenced by our group^37^. The sequenced MN transcriptome of five developmental stages namely the leaf, root, bark, male flower, and winter bud were downloaded from MorusDB^27^. A *de novo* transcriptome assembly software, Trinity (version r21040413p1)^38^, with default parameters was used to assemble a reference transcriptome from the seven developmental stages. A pre-assembly quality check was performed for these reads using Fastqc version 0.11.2^39^. Trimmomatic (version 0.30)^40^ was used to remove adapter contamination and primer sequences (partial or full), with the following parameters: LEADING: 5 TRAILING: 5 and MINLEN: 36. *In silico* normalization was also performed using Trinity. Assembled sequences were annotated using the FastAnnotator server^41^ from Chan Gung University, Taiwan. The assembled transcriptome from the seven stages was used as a reference to estimate abundance. A perl script align_and_estimate_abundance.pl, which is based on bowtie^42^, provided in the Trinity software was used to this end. After the estimation of abundance, expression values were generated using edgeR package of R^43^. A heat-map was generated to show the differential expression pattern of these genes based on the RSEM package of R^44^.

### Stress and hormonal treatments and expression profiling using qPCR

For qPCR experiments, *Morus indica* (MI) leaves were obtained from field grown plants from the gardens of Department of Plant Molecular Biology and Department of Botany, University of Delhi, (New Delhi). All leaves were at the same developmental stage. Control 0 h represents samples that were immediately frozen in liquid nitrogen, while control 4 h represents samples that were kept in reverse osmosed water for 4 h at 28 °C. To subject the samples to heat and cold stress, the setup was kept at 42 and 0 °C, respectively. Samples were subjected to salinity and drought stresses by transferring the setup to 250 mM NaCl and 0.5 M Mannitol solution, respectively, at 28 °C for 4 h. For hormonal treatment, the samples were treated with 100 μM solution of Jasmonic Acid, Salicylic Acid, Abscisic Acid, and Indole Acetic Acid (Sigma-Aldrich, Germany) for 4 h at 28 °C. For Epibrassinolide, 50 μM solution was used at 28 °C for 4 hrs. After subjecting the samples to the abiotic stresses and hormonal treatment, tissues were harvested, snap-frozen in liquid nitrogen and stored at −80 °C for later use. Total RNA from these samples were isolated using the GITC protocol^45^, with minor modifications. Total RNA was purified using the Rneasy Plant Mini Kit (Qiagen, Germany) and DNase treatment was given on the same columns. The RNA quality was checked using the NanoVue (GE Healthcare) and on a denaturing agarose gel. First strand cDNA was synthesized using 2.0 μg of total RNA in a reaction volume of 50 μl, using the high-capacity cDNA archive kit (Applied Biosystems, USA). The primers were designed with the Primer Express software version 2.0 (Applied Biosystems, USA) using default parameters. Uniqueness of the primers was checked by BLAST searches against the mulberry genome and melting curve analysis post PCR completion. PCR was performed using the ABI Prism 7000 sequence detection system (Applied Biosystems, USA). Two biological replicates with three technical replicates were taken for each sample. The data were normalized using Ct values obtained for Actin amplification. Relative expression levels were calculated using the ΔΔCT method. Finally, the mean ∂Ct values were statistically analyze using the Student’s t-test to identify the expression profiles showing significant changes. p ≤ 0.05 was considered statistically significant.

## Results

### Identification of WRKY gene family in mulberry

**Figure 1:**
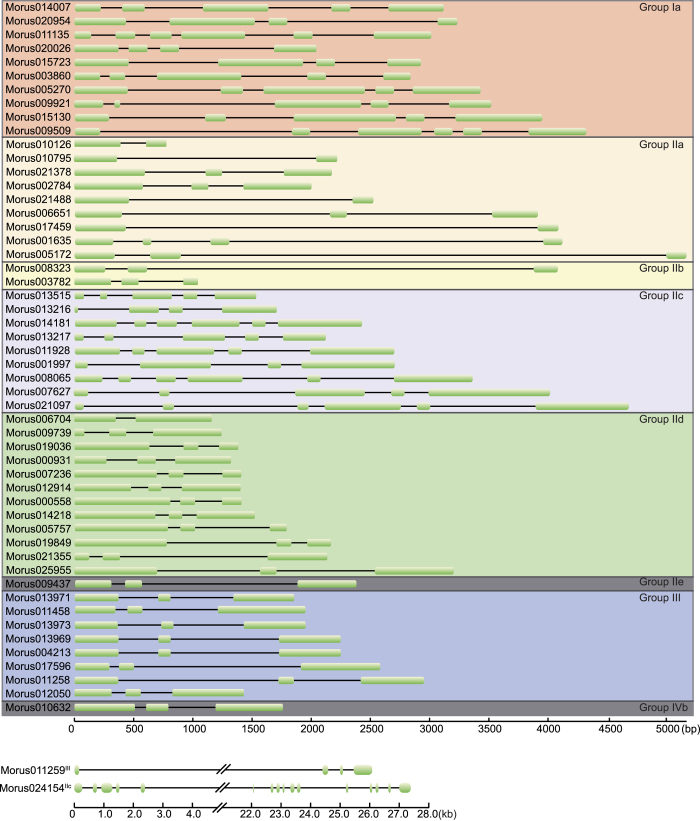
Illustration of the gene structure of 54 mulberry WRKY genes, showing the distribution of exons and introns (to map scale). Genes are separated into their respective groups. Exons are shown using solid boxes while introns are shown using solid lines. For greater resolution, two genes >5 kb in length are shown on a separate scale.

In total, 54 WRKY genes were identified in the MN genome using hmm and blast search methods. All of the identified WRKY genes showed the presence of the conserved WRKY domain in NCBI CDD and ScanProsite searches. Structural analysis of these 54 WRKY genes revealed that most of the genes were up to 5000 bp in length (Supplementary Table S1). Morus011259 and Morus024154 were 26.14 and 28.30 kb in size, respectively, and possessing very large introns (Fig. 1). WRKY mRNAs in mulberry ranged from 554 to 2357 bp. Twenty-seven genes possessed three exons and two introns followed by nine genes with five exons and four introns. Morus024154 had the maximum number of exons i.e. 16. The greatest exon length was found to be 1025 bp. The molecular mass of these proteins ranged from 16.33 to 64.91 kDa. Prediction of alternate spliced forms using GENSCAN^46^,^47^ revealed that 50 WRKYs encode single-spliced forms, while Morus009921 and Morus017459 encode two spliced forms. Morus024154 (Group IIc member) was found to encode for six spliced forms, whereas Morus011259 was found to encode five splice variants. These genes showed a wide range of isoelectric focusing points ranging from very acidic i.e., 4.66 to very basic i.e., 10.22 pKa, suggesting a wide range of occupancy in different microcellular environments. Thirty-one of 54 WRKYs showed an acidic pKa range, while the remaining showed had a basic pKa range. The hallmark of all WRKY protein is a conserved N terminal ‘WRKYGQK’ motif. This motif was found in all mulberry species investigated in this study. Several variants of this conserved WRKY motif have been reported in plants. In mulberry, two variants of this motif are found, ‘WRKYGQK’ and ‘WRKYGKK’. Majority of these proteins contain the ‘WRKYGQK’ motif, while ‘WRKYGKK’ is found in GroupIIb members, Morus008323 and Morus003782, which are the only members of this group (Supplementary Figure S1).

### Phylogenetic analysis of mulberry WRKYs

**Figure 2:**
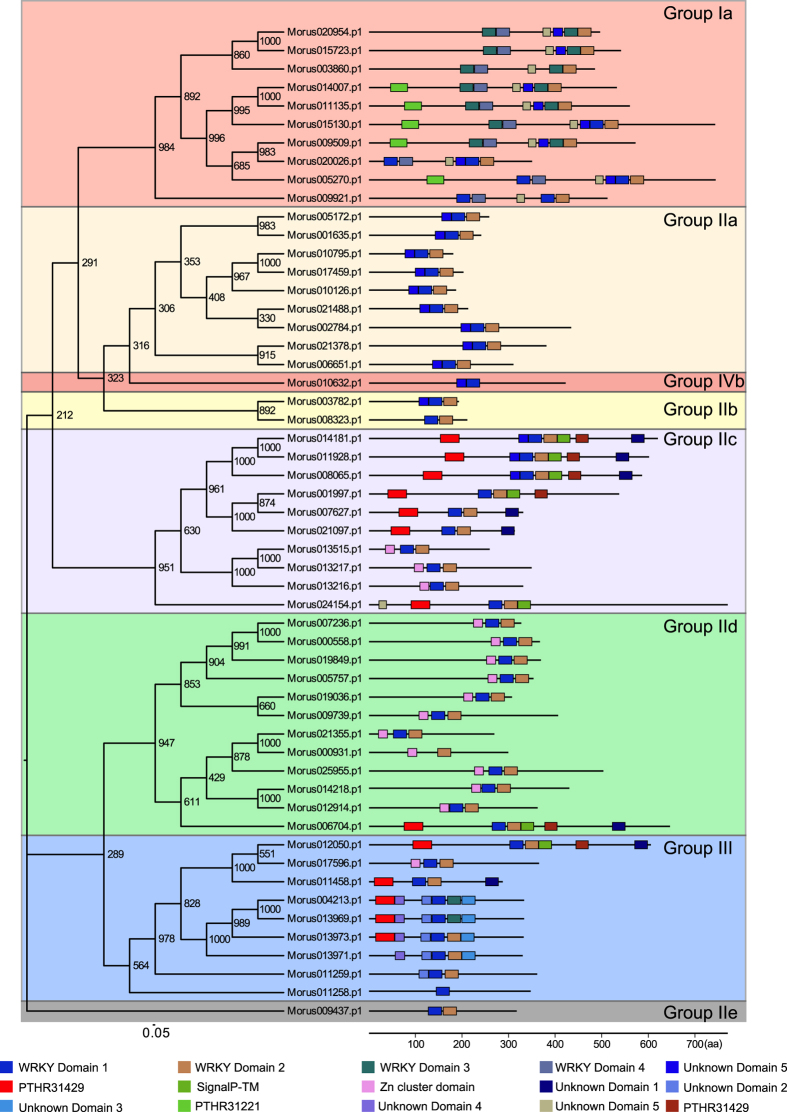
Phylogenetic tree depicting WRKY evolution in mulberry. Clades are highlighted using background colors. Proteins are shown with the predicted MEME motifs (to map scale). Motif annotation is shown as color legends.

Phylogenetic analysis of mulberry WRKYs revealed the presence of four gene groups. Classification of these WRKYs groups was based on the combination of WRKY domains and two different types of Zinc Finger motifs^2^. Group I members have two WRKY domains and two C_2_H_2_ signature motifs. Group I is further classified into Ia and Ib. Group Ib genes (with 2 WRKY domain + 2 C_2_HC motifs)^2^ in mulberry were not found in this study. In *Lotus japonicas*, which is also a member of same rosales evolutionary clade, no sub-group of group I members was reported^48^. All 10 members of group Ia form a distinct clade (Fig. 2). Moreover, at the C-terminal end of all Group Ia members, a conserved unknown domain with a signature motif (GGDFDDNEPEAKRWKGE) was found. This motif was also present in a member of Group IIb (Morus024154) but was present at the N-terminal. MEME motif search analysis revealed the presence of the SignalP-TM motif in five members of this clade namely Morus140007, Morus011135, Morus015130, Morus009509, and Morus005270. Group II members have one WRKY domain and one C_2_H_2_ motif and based on the cladistic arrangement, Group II was categorized into five subgroups (a–e). Mulberry was represented in all five sub-groups and Group IIa has 12 members without any additional domain. Groups IIb, IIc, IId and IIe have two, 10, 12, and one members, respectively. Group IId has 12 members in total and 11 of them possess the Zn cluster domain. Group III members have one WRKY domain and one C_2_HC motif. In mulberry, there are nine members in Group III. Group IV genes are proposed to have only a WRKY domain and they did not possess any Zinc Finger motifs. MEME motif analysis revealed additional WRKY domains in these sequences. Group IV in mulberry is represented by single gene, Morus010632. To identify additional domains, conserved pfam domain profiles were used and a total of 39 different conserved motifs were identified (Supplementary Figure S2). The most abundant domain identified was FAR1 DNA-binding domain (PF03101.10), which was expected because it contains a WRKY-like fold. The other domains with considerable representation include Zn_ribbon_recom (PF13408.1), Ogr_Delta (PF04606.7) and FLYWCH (PF04500.11), all of which are related to the zinc finger-binding domains. All but two members (Morus009509 and Morus009921) of Group Ia contain the FAR1 (PF03101.10) conserved domain in their protein sequences. A supplementary file containing the details of the pfam domain distribution in these proteins has been provided (Supplementary Table S2).

### Upstream regulatory regions and *cis*-elements

**Figure 3:**
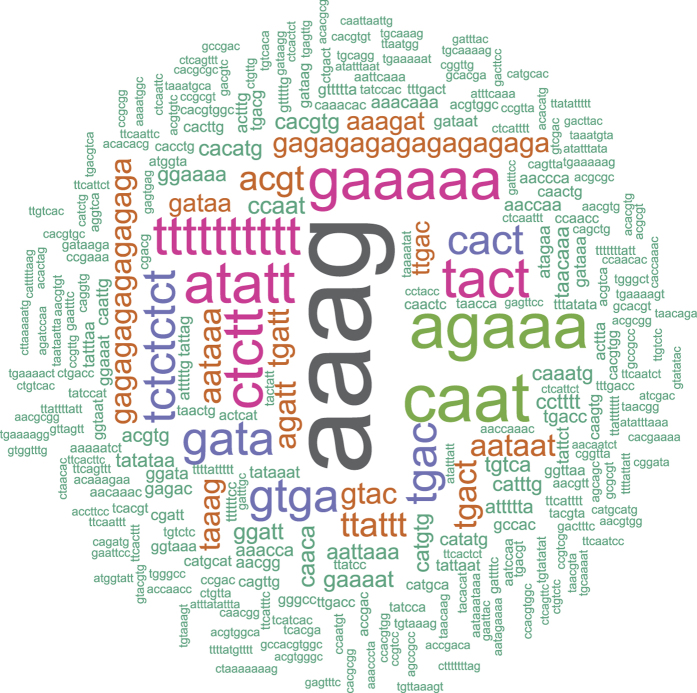
Word-cloud showing the identified *cis* elements in the putative promoter regions of WRKY genes in mulberry. The intensity and size of the motifs indicate their frequency.

Of the 54 genes, 2K upstream regulatory region (promoter) could be fetched in 52. The promoter of Morus013969 could not be isolated because it lies to the extreme left of the assembled scaffold to which it belongs. Moreover, only 1085 promoter bases of the Morus001635 could be fetched as only these many bases are available upstream in the scaffold. ‘Word clouds’ or ‘TAG clouds’ are the presentation of words selected by rationale of being represented more frequently. A word cloud for all the *cis* motifs in mulberry WRKY promoters were generated (Fig. 3). This word cloud revealed the frequent presence of motifs such as, AAAG, GAAAA, AGAAA. In total, 14 important motifs were scanned (Supplementary Figure S3; Supplementary Table S3). The five most-frequent motifs included the GT-1 motif (GAAAAA; responsible for pathogen- and salt-induced expression), W Box (TTGAC; recognized specifically by salicylic acid (SA)-induced WRKY DNA-binding proteins), ASF-1 binding site (TGACG; involved in transcriptional activation by auxin and salicylic acid treatment), Low temperature responsive elements (CCGAAA, ACCGACA and CCGAC), and ARF binding site (TGTCTC; Auxin Response Factor Binding site). ARF binding sites^49^ were present in 25 genes and were represented 41 times. AuxRE (Auxin response element; KGTCCCAT) was present in the promoter of two genes namely Morus009921 and Morus021355. Morus009921 harbors two such elements in its promoter. Dreb binding site (TACCGACAT) was present in the promoter of 23 genes and was represented 36 times. TCA-1 motif (TCATCTTCTT), responsible for bringing the salicylic acid mediated gene expression, was found in the promoter of Morus011259. ABA responsive element was found in the promoter of 12 genes and it was represented 17 times. The core of GCC Box (GCCGCC; shown to regulate jasmonic acid-induced expression) was found in the promoter of 16 genes in total. The T/G-box (AACGTG; involved in jasmonate-induced genes via JAMYC2 and 10) was found in the promoters of 23 WRKYs and was represented 39 times.

### GO enrichment analysis of WRKY genes in mulberry

GO terms are the descriptions of the gene products and are structured around three ontologies that describe the molecular function, sub-cellular component distribution and involvement in the biological processes of a gene. In GO enrichment analysis, in terms of biological processes, the top five significant terms were regulation of transcription (GO:0045449), regulation of transcription, DNA-dependent (GO:0006355) regulation of RNA metabolic process (GO:0051252), regulation of nucleobase, nucleoside, nucleotide and nucleic acid metabolic process (GO:0019219), and regulation of nitrogen compound metabolic process (GO:0051171) (Supplementary Figure S4 and Supplementary Table S4). Besides, the respiratory burst involved in defense response (GO:0002679), regulation of defense response to virus by host (GO:0050691), salicylic acid-mediated signaling pathway (GO:0009863), phytoalexin biosynthetic process (GO:0052315), response to jasmonic acid stimulus (GO:0009753), MAPKKK cascade (GO:0000165), cellular response to endogenous stimulus (GO:0071495), signal transduction (GO:0007165), cellular response to stress (GO:0033554), response to external stimulus (GO:0009605), embryonic pattern specification (GO:0009880), embryonic axis specification (GO:0000578), and response to absence of light (GO:0009646) were the other significantly enriched biological process GO terms. This indicates their putative role in defense response, developmental aspects, and stress-coping mechanisms.

In cellular components, the top five significant GO terms included, transcription factor complex (GO:0005667), nucleoplasm part (GO:0044451), nucleoplasm (GO:0005654), nuclear lumen (GO:0031981), and nuclear part (GO:0044428), suggesting their role in regulation of transcription machinery (Supplementary Figure S5(A); Supplementary Table S5). Moreover, the DASH complex (GO:0042729) and condensed nuclear chromosome, centromeric region (GO:0000780) terms were also found to be significant, suggesting their putative role in spindle-attachment, chromosome segregation and spindle stability.

In GO molecular function, sequence-specific DNA binding (GO:0043565), transcription factor activity (GO:0003700), and transcription regulator activity (GO:0030528) terms were significantly highlighted (Supplementary Figure S5(B); Supplementary Table S6). Further, this ontological category also included calmodulin binding (GO:0005516), phospholipid-translocating ATPase activity (GO:0004012), phospholipid transporter activity (GO:0005548), and lipid transporter activity (GO:0005319) terms, suggesting their participation in diverse range of signaling cascades.

### Expression pattern in various developmental stages

**Figure 4:**
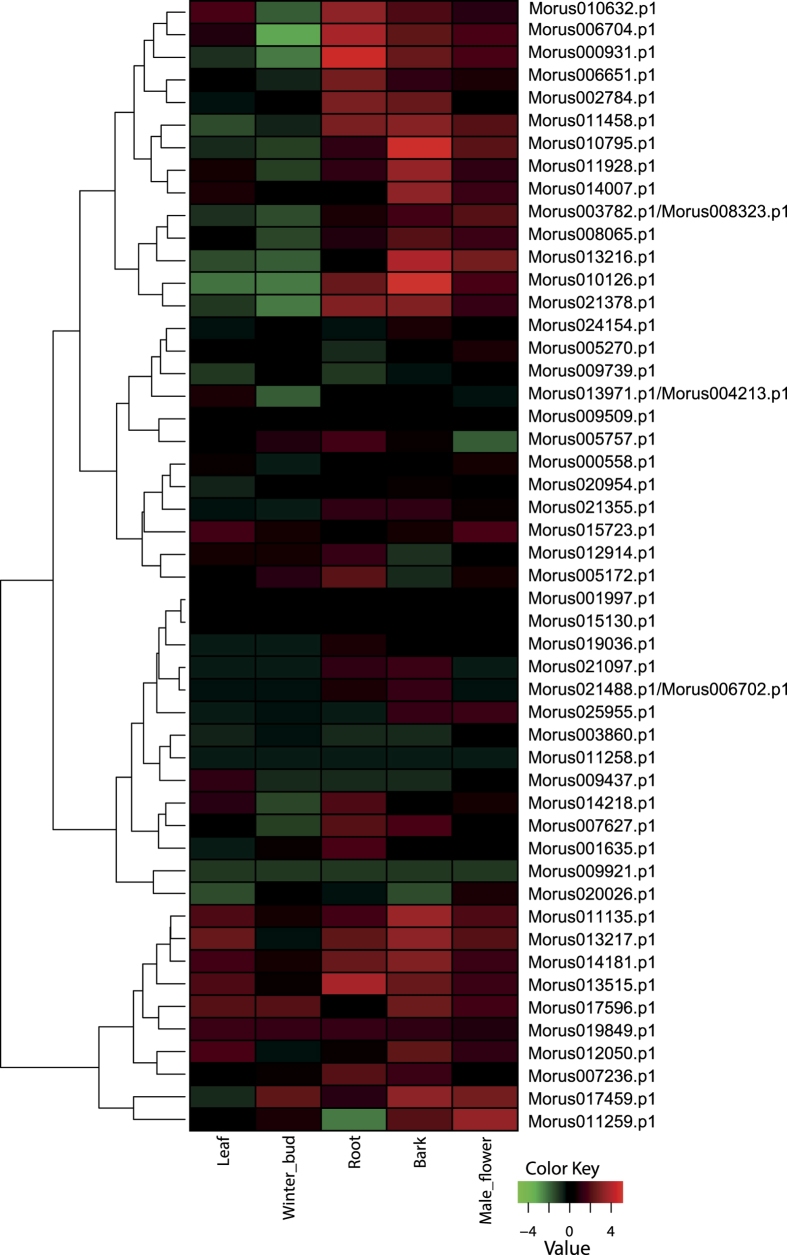
Heat map showing the expression pattern of WRKY genes across five developmental stages. Heat map was generate using TMM-normalized FPKM values obtained for the respective stages. Legend shows the level of up- or down-regulation.

Expression analysis of WRKY genes in five developmental stages, namely the leaf, root, bark, male flower, and winter bud revealed a wide range of expression patterns across the various stages. Expression profiles for 50 mulberry WRKY genes were ascertained by RNA-seq analysis (Fig. 4). Thirteen WRKY genes were found to have the highest expression in MN root tissue. A maximum of 25 WRKYs were found to have the highest expression in the bark tissue. None of the WRKYs showed preferential expression in the winter bud, while the male flower had a total of 10 WRKY genes, having the highest expression compared to other stages. Morus107459,which has *cis-*elements conferring salicylic acid responsiveness in its promoter, had the highest expression in the bark tissue.

### Comparative expression profiles in the three mulberry species

**Figure 5:**
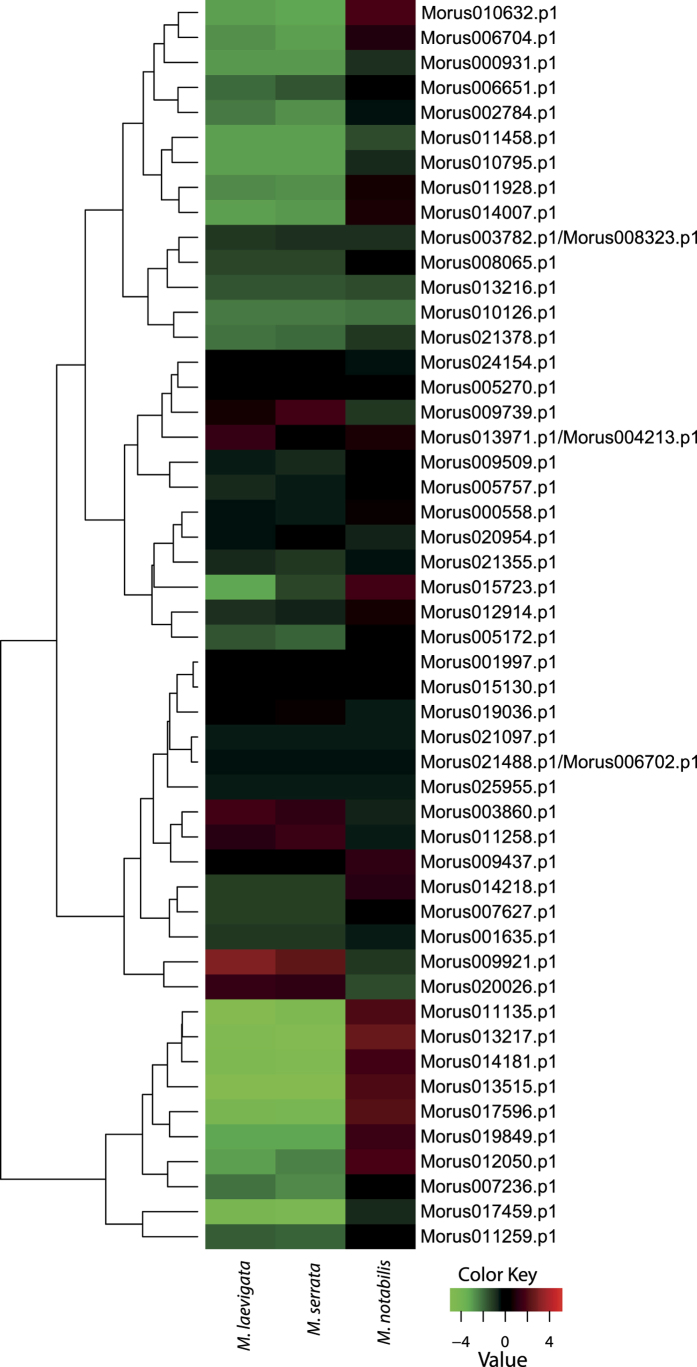
Heat map showing the comparative expression level of WRKY genes in three genotypes i.e., *Morus laevigata, Morus serrata*, and *Morus notabilis*. Genes with comparatively lower expression values are shown using shades of green and high expression values are represented using shades of red. TMM-normalized FPKM values were used to generate the heat map.

Mulberry leaf is most important tissue because it is the sole source of feed for the silkworm. Silk yield depends on the quality and quantity of leaves. MN, MS, and ML are proposed to have a diverse range of resistance genes^23^,^50^. These species are believed to cater to the need for mulberry improvement by virtue of having diverse genetic backgrounds with a robust gene pool. Therefore, to identify the role of WRKY in these genotypes, we compared the expression patterns of these genes. Of the 50 WRKY genes identified from the assembled transcriptome, 20 showed the highest expression in MN (Fig. 5), followed by 16 in ML, and 14 in MS. A cluster of 10 genes showed very high expression in MN and relatively much lower expression in ML and MS. Morus011135, Morus013217, Morus014181, Morus013515, Morus017596, Morus019849, Morus012050, Morus007236, Morus017459 and Morus011259. Morus009921, Morus020026, and Morus003860 showed the highest expression in ML, while Morus0099739, Morus011258 showed the highest expression in MS.

### Differential regulation of Morus WRKYs in response to stress and hormonal treatments

**Figure 6:**
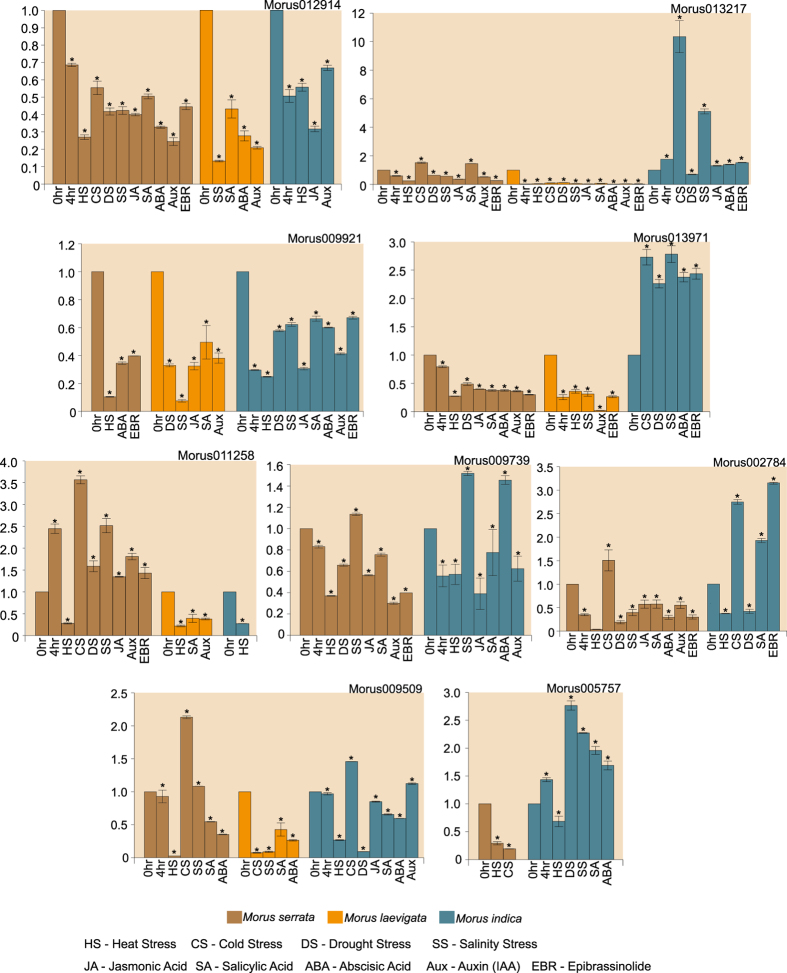
Bar graphs showing the relative fold change of selected WRKY genes in different mulberry species. Error bars depict standard error for two biological replicates. Three technical replicates for each biological replicates were run. Relative fold changes were obtained using the ∂∂Ct method. Asterisks on top of the error bars represent the significance levels (Students *t*-test; p value ≤ 0.05). See legend for details.

Nine WRKYs out of 54 were selected for further analysis based on their genotype-preferential expression patterns in the leaf tissue. Two genes, each showing relatively higher expression in ML and MS, and five genes showing higher expression in MN were chosen. The expression patterns of these selected genes were investigated in three genotypes including ML, MS and *Morus indica* (MI) in response to various stress and hormone treatments. The primer pairs used to amplify the respective transcripts are presented in Supplementary Table S7. Different WRKY genes were differentially expressed in response to various treatments (Fig. 6).

Three genes namely Morus009739, Morus002784 and Morus005757 did not show significant differential expression in response to the given treatments in ML. Morus01294 was found to be downregulated significantly in response to various treatments in all three genotypes studied. However, these responses differed for different treatments. Morus013217 showed a more than 10- and 5-folds up-regulation in MI in response to cold and salinity stress, respectively. The same gene was found to be down-regulated in response to all the treatments in ML. Another identified WRKY (Morus009921) was also found to be down-regulated in response to various treatments in all the three genotypes. The maximum reduction in the expression was found in heat stress-treated MS and salt stress-treated ML. Morus013971 was found to be down-regulated in all the treatments in MS and ML, while the same gene was up-regulated in MI in cold, dehydration, salt, ABA and EBR treatments. Morus011258 was up-regulated in MS in response to every treatment except heat stress. The same gene was down-regulated in other two genotypes, ML and MI. Morus009739 was expressed differentially in only two genotypes in various treatments. Similarly, Morus002784 was also up-regulated in response to cold stress, salicylic acid and EBR treatment in MI. Up-regulation of Morus009509 was observed in MS and MI in response to cold treatment. This gene was downregulated in ML and the most severe reduction in expression in this genotype was reported under cold and salt stresses. Morus005757 was up-regulated in MI only and its expression was downregulated under heat stress in same genotype.

## Discussion

Mulberry is the most important tree species for the silk industry as >90% of the world’s silk production depends on it. Its leaves are the only source of food for the monophagous silk worm, *Bombyx mori*. Despite its importance, research on mulberry is limited. Recently, the successful genome sequencing of a wild relative of mulberry has sparked great interest in the mulberry research community. Mulberry is susceptible to various abiotic and biotic stresses. Conventional breeding efforts have relied on wild varieties to integrate important genes to combat these stresses. WRKY genes have been shown to be a versatile regulator of various kinds of stresses; however, this important gene family has not been characterized in mulberry thus far. In this study, 54 WRKY-encoding genes were identified from the MN genome. Seventy-four and 109 members were identified in *Arabidopsis* and *Oryza sativa*, respectively^2^,^51^, while 55 members have been identified in *Cucmis sativus*^52^ and 95 in *Daucus carota*^53^. Based on the sequence similarity and distribution of conserved domains, WRKYs in mulberry were divided into four groups. We observed a substantial huge variation in terms of protein isoelectric focusing point, exon intron distribution, and molecular mass. Further, 38 different types of domains were found in these WRKYs, suggesting that in order to perform additional functions these WRKYs have acquired additional domains, which leads to variations in their structural and physiological features. Similar to another Rosid member, *Lotus*^48^, Group 1b was not found in mulberry, suggesting that this group is absent in Rosids. Analysis of this gene family in other members of Rosales might shed light on the evolutionary pattern of WRKY Group Ib members. WRKY motif was found to be fairly conserved in mulberry as only two variants of this motif were found in this study. All the members except Group IIb possessed ‘WRKYGQK’ while GroupIIb members of mulberry possess the second variant ‘WRKYGKK’. Compared to these findings, nine variants of the WRKY motif have been reported in rice^54^. We also found that a conserved small domain of unknown function was found in all Group 1a members, which might possess some hitherto uncharacterized role. Group IIc members of mulberry possess the maximum number of introns in their gene structure. Differential responses of several WRKYs are regulated by the presence of *cis* elements in their promoter region^55^,^56^. In total, 14 important *cis* elements and their distribution were studied. Three LTREs were found in the promoter regions of Morus013217, which showed a strong response to cold stress. This suggests that the identified LTRE might be functional in the promoter of this gene. This gene also showed up-regulation in response to salt stress. A motif (TACCGACAT) previously known to show responsiveness to drought, low temperature, and high salt stress was also found in the promoter of this gene. Morus012914 had also showed responsiveness to cold stress and its expression was up-regulated, and LTRE elements were found in its promoter. Morus005757 showed a more than two fold up-regulation in response to SA treatment. In the promoter of this gene, ASF-1 binding site and four W-Boxes variants were found, which are recognized by the SA-induced WRKY genes. This suggests a possibility that Morus005757 might be regulated by other WRKYs, which are induced by SA treatment. Morus011258 was up-regulated in response to auxin treatment. Although it does not contain any AUXRE, two ARF-binding sites were found in the upstream region of this gene, indicating that some early auxin-induced ARF might regulate the expression of this gene. Morus005757 has also showed positive regulation in response to auxin treatment but no ARF or AUXRE are found in the promoter of this gene. However, the promoter of this gene contains an ASF-1 element. Therefore, its auxin-mediated induction might be regulated using the ASF-1 binding site, which is also known to respond to auxin treatments. In transcriptome studies, the expression pattern of these WRKY genes were identified in five developmental stages. Our analysis has revealed the tissue-specific expression exhibited by certain members. Thirteen of 54 identified WRKYS showed preferential accumulation in the root tissue. In *Salvia miltiorrhiza*, 22 out of 61 identified WRKYs have been found to show preferential expression in root tissue^57^. OsWRKY74, which has a root preferential expression, has a proven role in the phosphate-starvation tolerance^58^. We found 25 WRKYs to be expressed preferentially in bark tissue. In a previous study, the putative role of WRKYs in bark component synthesis was found^59^, indicating a possible involvement of these WRKYs in the synthesis of bioactive constituents in the bark tissue of mulberry. In *Ginkgo biloba*, out of 28 identified WRKYs, 20 showed relatively higher accumulation in the male flower, and GbWRKY2 showed preferential accumulation in the male and female flowers^60^. In our study, we identified 10 WRKYs showing preferential accumulation in the male flower. Among the three genotypes studied, 20 WRKYs were found to have relatively higher expression in MN. Sixteen and 14 WRKYs have showed comparatively higher expression in ML and MS than in MN, respectively. These genotypes are reported to harbor numerous SNP/INDELS in the coding regions of their transcriptome^37^. The expression pattern exhibited by these genotypes is possibly the result of such variations in the gene as well as promoter regions. Considering the wider adaptability and resilience exhibited by ML compared to MS, these differentially expressing WRKYs might play some role in their adaptation.

## Conclusion

The WRKY catalogue of mulberry was identified to possess a total of 54 genes. Structural and functional analysis revealed the presence of representatives of all but Group Ib members in mulberry, suggesting a possible absence of Group Ib members in the Rosids. This could be further confirmed in other Rosales members. Analysis of *cis* elements in 2K upstream region revealed the presence of several important motifs pertaining to WRKY functions under biotic and abiotic stresses. An uncharacterized, conserved domain specific to Group Ia members was identified, which could have some evolutionary significance. Gene ontology enrichment analysis had revealed a broader range of molecular functions, biological processes, and cellular component distribution. WRKYs in mulberry showed a wide range of expression profiles and were found to express in a tissue-specific manner. Three mulberry species, in which expression profiles WRKY genes were compared, belonged to different agro-climatic conditions. These species differ in their leaf morphology and are considered as potential sources of resistance genes^23^,^50^. These WRKYs might likely be the causative factors for their local adaptations. During qPCR analysis, selected WRKYs showed modulation in response to stress and hormonal treatments, and this response was specific to some genotypes in relation to certain WRKY genes. Morus013217 and Morus002784 showed higher accumulation in response to cold stress and salt stress. Thus, these genes can be further selected for functional validation in response to cold and salt stress. Morus005757 showed significant up-regulation in response to dehydration stress, salinity stress, and salicylic acid and ABA treatment. Thus, this gene could be further characterized. Taken together, our study has generated an important resource that could be used for further studies of WRKY genes in mulberry and could be used as a cue for varietal improvements.

## Additional Information

**How to cite this article**: Baranwal, V. K. *et al*. Genome-wide Identification and Structural, Functional and Evolutionary Analysis of WRKY Components of Mulberry. *Sci. Rep.* **6**, 30794; doi: 10.1038/srep30794 (2016).

## Supplementary Material

Supplementary Information
