# Supplementary material for: Genome-wide Identification and Structural, Functional and Evolutionary Analysis of WRKY Components of Mulberry
Source: Sci Rep. 2016 Aug 1;6:30794. doi: 10.1038/srep30794 (PMC4967854; doi:10.1038/srep30794)
Supplement: Supplementary Information [file srep30794-s1.pdf]

Genome-wide Identification and Structural, Functional and Evolutionary Analysis of WRKY Components of Mulberry

Vinay Kumar Baranwal[1], Nisha Negi[1] and Paramjit Khurana[1*]

[1] Department of Plant Molecular Biology, University of Delhi South Campus, Benito Juarez Road, New Delhi, 110021, India

[*]Corresponding Author email: param@genomeindia.org

Supplementary Figure S1. Alignment of WRKY domains of the identified members of mulberry showing the conserved WRKY motifs.

```
                                  *   *****:*
Morus007236.p1_254-311 -----PDDYSWRKYGQKPIKGSPHPRGYYKCSSVR--GCPARKHVERALDDPSMLVVTYEGEHNH--   58
Morus005757.p1_285-342 -----PDDYSWRKYGQKPIKGSPHPRGYYKCSSMR--GCPARKHVERCLEDPSMLIVTYEGEHNH--   58
Morus000558.p1_291-349 ----PPDEYSWRKYGQKPIKGSPHPRGYYKCSSLR--GCPARKHVERALDDPAMLIVTYEGDHNH--   59
Morus019849.p1_281-339 ----PQDEYSWRKYGQKPIKGSPYPRGYYKCSTMR--GCPARKHVERAPDDPAMLIVTYEGEHRH--   59
Morus019036.p1_233-290 -----PDDYSWRKYGQKPIKGSPYPRSYYKCSSVK--GCPARKHVERCLDDPTMLVVTYEGDHNH--   58
Morus025955.p1_260-318 ----PSDLWAWRKYGQKPIKGSPYPRGYYRCSSSK--GCSARKQVERSRTDPNMLVVTYTSEHNH--   59
Morus000931.p1_121-175 ----PSDSWAWRKYGQKPIKGSPYPR----CSTSK--GCPARKQVERSRVDPTMLVVTYSAEHNH--   55
Morus021355.p1_70-128  ----PSDSWAWRKYGQKPIKGSPYPRGYYRCSSSK--GCPARKQVERSRVDPTTLIITYACNHNH--   59
Morus012914.p1_178-234 ------DIWAWRKYGQKPIKGSPYPRGYYRCSSSK--GCLARKQVERNRSDPGMFIVTYTAEHNH--   57
Morus006704.p1_130-192 EDGLCADMWAWRKYGQKPIKGSPYPRSYYRCSSSK--GCSARKQVERSPSNPEIFVITYTAEHSH--   63
Morus014218.p1_247-303 ------DLWAWRKYGQKPIKGSPYPRNYYRCSSSK--GCAARKQVERSNVDPDLFIVTYTGEHTH--   57
Morus009739.p1_55-113  ----PSDFWSWRKYGQKPIKGSPYPRGYYRCSTSK--GCSAKKQVERCKTDDSLLIITYTSSHNH--   59
Morus020954.p1_243-301 ----ADDGYNWRKYGQKQVKGSEFPRSYYKCTHL---NCPVKKKVER-SPDGQITEIIYKGQHNHEK   59
Morus015723.p1_246-301 -----DDGYNWRKYGQKQVKGSEFPRSYYKCTHP---NCPVKKKVER-SLEGQVTEIIYKGQHNH--   56
Morus014007.p1_195-253 ----SDDGYNWRKYGQKQVKGSENPRSYYKCTNP---NCPMKKKVER-SLDGQITEIVYKGSHNHPK   59
Morus011135.p1_207-265 ----SDDGYNWRKYGQKQVKGSENPRSYYKCTYP---NCPTKKKVER-ALDGQITEIVYKGSHNHPK   59
Morus003860.p1_198-254 ------DGYNWRKYGQKQVKGSEFPRSYYKCTHP---NCPVKKKVER-SLDGQIAEIVYKGEHNHSK   57
Morus015130.p1_257-315 ----SEDGYNWRKYGQKQVKGSEYPRSYYKCTHT---DCQVKKKVER-SHDGQITEIIYKGSHNHAK   59
Morus005270.p1_320-378 ----SEDGYNWRKYGQKQVKGSEYPRSYYKCTHL---NCQVKKKVER-SHEGHITEIIYKGSHNHPK   59
Morus009509.p1_215-273 ----SDDGYNWRKYGQKHVKGSEFPRSYYKCTHP---NCEVKKLFER-SHDGQITEIIYKGTHDHPK   59
Morus020026.p1_37-93   ------DGYNWRKYGQKLVKGNEYVRSYYRCTHP---NCQVKKQLEC-SHDRQIVDIVYFGHHDHPK   57
Morus015130.p1_477-535 ----LDDGYRWRKYGQKVVKGNPNPRSYYKCTSA---GCSVRKHVERASHNLKFVITTYEGKHNHE-   59
Morus005270.p1_532-590 ----LDDGYRWRKYGQKVVKGNPNPRSYYKCTNA---GCTVRKHVERASHDLKSVITTYEGKHNHD-   59
Morus014007.p1_355-413 ----LDDGYRWRKYGQKVVKGNPNPRSYYKCTSV---GCPVRKHVERASHDTRAVITTYEGKHNHD-   59
Morus011135.p1_377-435 ----LDDGYRWRKYGQKVVKGNPNPRSYYKCTNP---GCPVRKHVERASHDLRAVITTYEGKHNHD-   59
Morus009509.p1_388-446 ----LDDGYRWRKYGQKVVRGNPNPRSYYKCTNA---GCPVRKHVERASHDPKAVITTYEGKHNHD-   59
Morus020954.p1_419-477 ----LDDGYRWRKYGQKVVKGNPHPRSYYKCTNA---GCNVRKHVERASTDPKAVITTYEGKHNHD-   59
Morus015723.p1_425-483 ----LDDGYRWRKYGQKVVKGNPYPRSYYKCTTT---GCNVRKHVERASSDSKAVITTYEGKHNHD-   59
Morus003860.p1_389-446 ------DGFRWRKYGQKVVKGSPYPRSYYRCTSL---KCNVRKHVERASDDPRAFITTYEGRHNHDM   58
Morus009921.p1_374-431 ------DGYRWRKYGQKMVKGNPHPRNYYRCTSA---GCPVRKHIETAVDNMNAVIITYKGIHDHDM   58
Morus020026.p1_210-267 ----VNDGHRWRKYGQKIVKGSPHPRSYYRCSNS---GCPVKKHVERASHDAKVVITTYEGEHNH--   58
Morus010795.p1_101-159 ----LDDGYRWRKYGQKAVKNNKFPRSYYRCTHQ---GCNVKKQVQRLTKDEGVVVTTYEGMHTHP-   59
Morus017459.p1_123-180 ----LDDGYRWRKYGQKAVKNNKFPRSYYRCTHQ---GCNVKKQVQRLTKDEGVVVTTYEGMHSH--   58
Morus010126.p1_109-168 ----LDDGYRWRKYGQKTVKNSKFPRSYYKCTYQ---GCNVKKQIQRHCKDEGIVVTTYEGMHTHPL   60
Morus021488.p1_133-192 ----LDDGYRWRKYGQKAVKNSSYPRSYYRCTHH---TCNVKKQVQRLSKDTSIVVTTYEGIHNHPC   60
Morus021378.p1_225-284 ----LEDGYRWRKYGQKAVKNSPYPRSYYRCTTQ---KCTVKKRVERSFQDPSIVITTYEGQHNHHC   60
Morus010632.p1_212-232 ----LEDGYRWRKYGQKAVKNSPYP------------------------------------------   21
Morus006651.p1_160-217 ----LEDGYRWRKYGQKAVKNSPFPRSYYRCTNS---KCIVKKRVERSSEDPTIVITTYEGQHCH--   58
Morus002784.p1_221-280 ----LDDGYRWRKYGQKAVKNNPYPRSYYRCTSG---RCGVKKRVERSSDDATIVVTTYEGQHTHPC   60
Morus005172.p1_180-239 ----LDDGYKWRKYGQKVVKNTQHPRSYYRCTQD---NCRVKKRVERLAEDPRMVITTYEGRHVHSP   60
Morus001635.p1_166-225 ----LDDGYKWRKYGQKVVKNSLHPRSYYRCTHN---NCRVKKRVERLSEDCRMVITTYEGRHNHSP   60
Morus003782.p1_131-188 ----LDDGFKWRKYGKKMVKNSPNPRNYYRCSVE---GCPVKKRVERDREDPRYVITTYEGVHNH--   58
Morus008323.p1_122-180 ----LDDGFRWRKYGKKSVKNSPNPRNYYKCSSG---GCNVKKRVERDRDDSSYVITTYEGVHNHE-   59
Morus008065.p1_327-385 ----LSDGCQWRKYGQKMAKGNPCPRAYYRCTMAT--GCPVRKQVQRCAEDQTILTTTYEGHHNH--   59
Morus011928.p1_328-384 ------DGCQWRKYGQKMAKGNPCPRAYYRCTMAA--GCPVRKQVQRCAEEQTILVTTYEGNHNH--   57
Morus014181.p1_347-403 ------DGCQWRKYGQKMAKGNPCPRAYYRCTMAV--GCPVRKQVQRCAEDRTILITTYEGNHNH--   57
Morus001997.p1_238-295 -----NDGCQWRKYGQKIAKGNPCPRAYYRCTVAP--SCPVRKQVQRCAEDMSILTTTYEGTHNH--   58
Morus021097.p1_306-363 -----NDGCQWRKYGQKIAKGNPCPRAYYRCTVSP--TCPVRKQVQRCAEDMSILITTYEGTHNH--   58
Morus007627.p1_268-325 -----NDGCQWRKYGQKIAKGNPCPRAYYRCTVAP--SCPVRKQVQRYADDISILITTYEGTHNH--   58
Morus024154.p1_260-318 ----LNDGCQWRKYGQKIAKGNPCPRAYYRCTVAP--GCPVRKQVQRCLEDMSILITTYEGNHNH--   59
Morus013515.p1_159-217 ----VKDGYQWRKYGQKVTRDNPSPRAYFKCSFAP--SCPVKKKVQRSVEDQSILVATYEGEHNH--   59
Morus013217.p1_173-231 ----VKDGYQWRKYGQKVTRDNPSPRAYFKCSFAP--CCPVKKKVQRSAEDPTLLVVTYEGEHSH--   59
Morus013216.p1_96-154  ----VKDGCQWRKYGQKVTKDNPFPRAYFRCSMAP--GCPVKKKVQRSMEDKSILVATYEGQHNH--   59
Morus009437.p1_130-187 ----ADDGYKWRKYGQKSIKNSPNPRSYYRCTNP---RCSAKKQVERSSDDPDTLIITYEGLHLH--   58
Morus011458.p1_137-197 ----LDDGFSWRKYGQKDILGAKYPRGYYRCTHRNVQGCLATKQVQRSDEDPNIFEITYRGRHTC--   61
Morus012050.p1_128-187 -----DDGYSWRKYGQKDILGAKYPRSYYRCTFRNTQSCWATKQVQRSDEDPYLFEITYRGTHTC--   60
Morus017596.p1_120-180 ----LEDGYSWRKYGQKDILGANHPRGYYRCTRRNAQGCLATKQVQRSDQDSSLFEVTYRGRHTC--   61
Morus004213.p1_139-198 -----DDGHAWRKYGQKMILKAKHPRHYYRCTNKYDQGCQATKQFQKIQDNPPIFRTTYCGHHTC--   60
Morus013969.p1_139-198 -----DDGHAWRKYGQKMILKAKHPRHYYRCTNKYDQGCQATKQFQKIQDNPPIFRTTYCGHHTC--   60
Morus013973.p1_137-196 -----DDGHAWRKYGQKMIIKGKHPRHYYRCTNKYDQGCQATKQVQKIQESPPIFRTTYCGHHIC--   60
Morus013971.p1_139-198 -----DDGHAWRKYGQKMILKGKHPRHYYRCTHKNDQGCQATKQVQKIQDNPPIFRTKYYGHHRC--   60
Morus011259.p1_131-191 ----LEDGQAWRKYGQKDILNAQYPRAYFRCTRKYDQGCRATKQVQQIQDNPKLYKITYIGQHTC--   61
Morus011258.p1_147-207 ----PDDGYTWRKYGQKEIMGSKYPRGYYRCTHQKLYDCPAKKQVQRLDHDPSTFEVIYHSHHTC--   61
                       1.......10........20........30........40........50........60.......
```

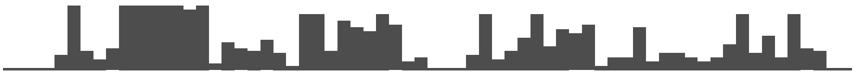

Supplementary Figure S2. Domains distribution in WRKY proteins of mulberry identified in hmmscan search against Pfam database.

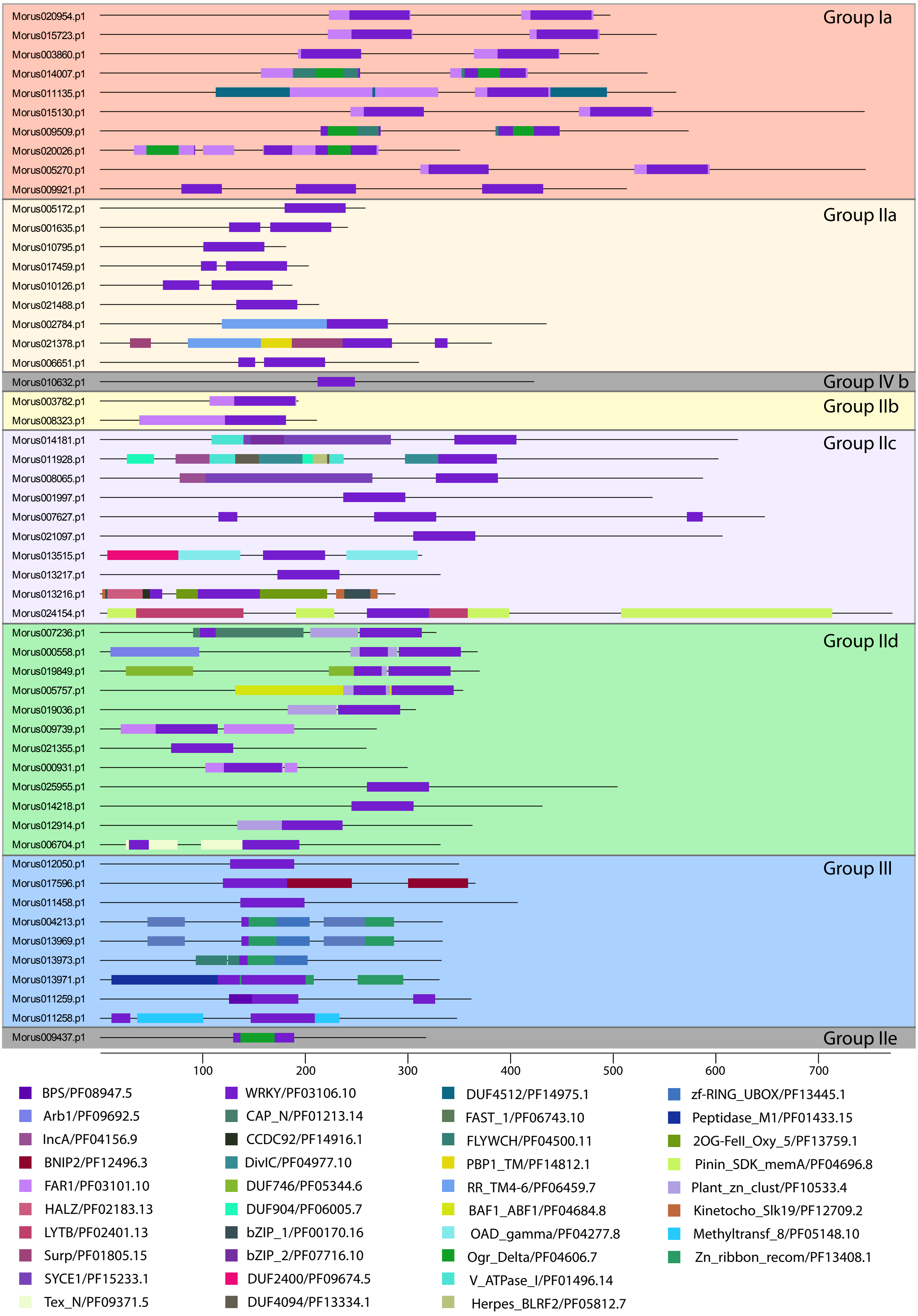

Supplementary Figure S3. Distribution of fourteen important cis elements identified in putative promoter region (2K Upstream) of WRKY genes (to scale). Colored legends are given to depict different cis elements.

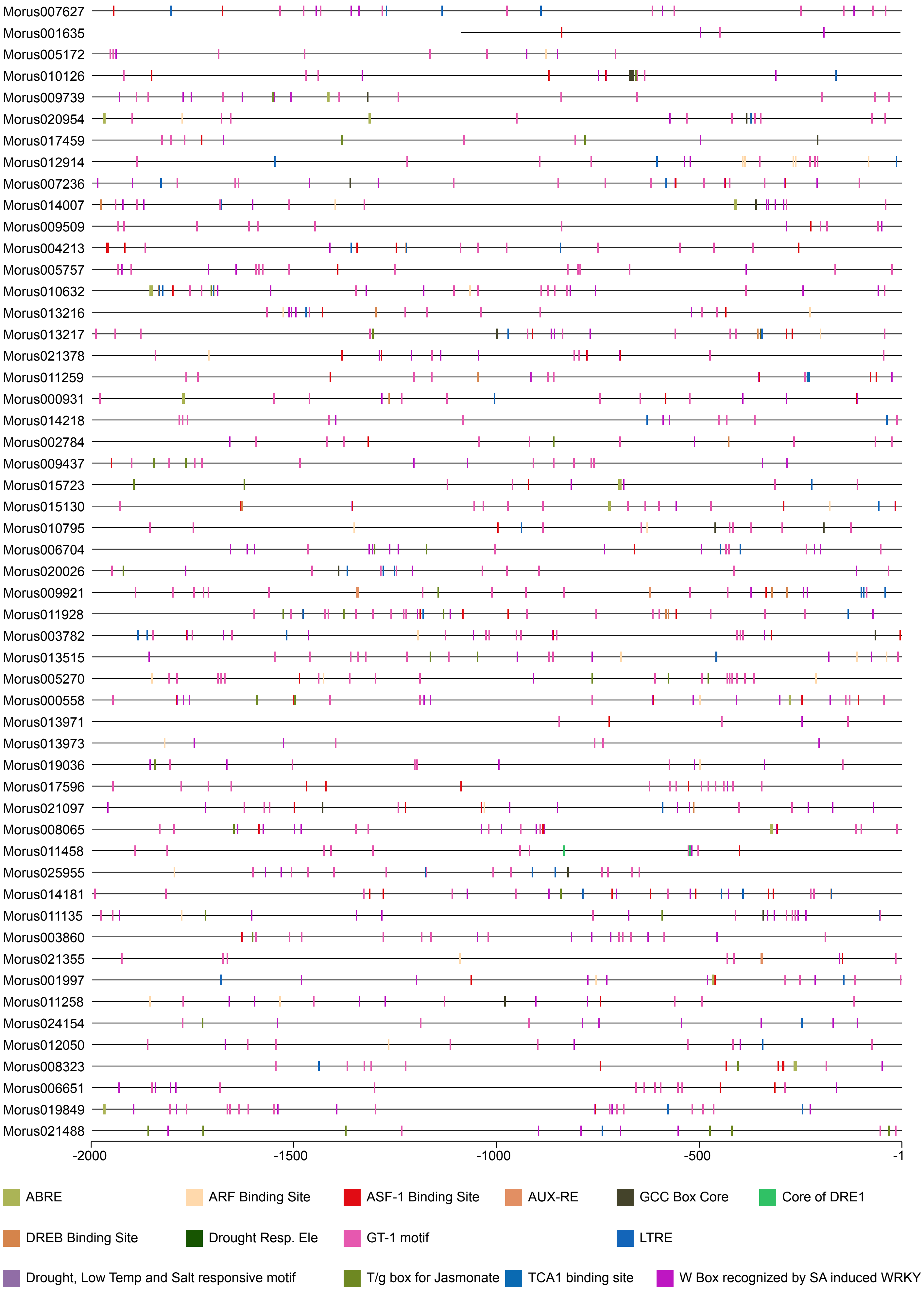

Supplementary Figure S4. Gene ontology terms enrichment analysis results are shown for WRKY genes in mulberry for Biological Process terms. Size of the nodes are representative of number of genes and color represents the significance level.

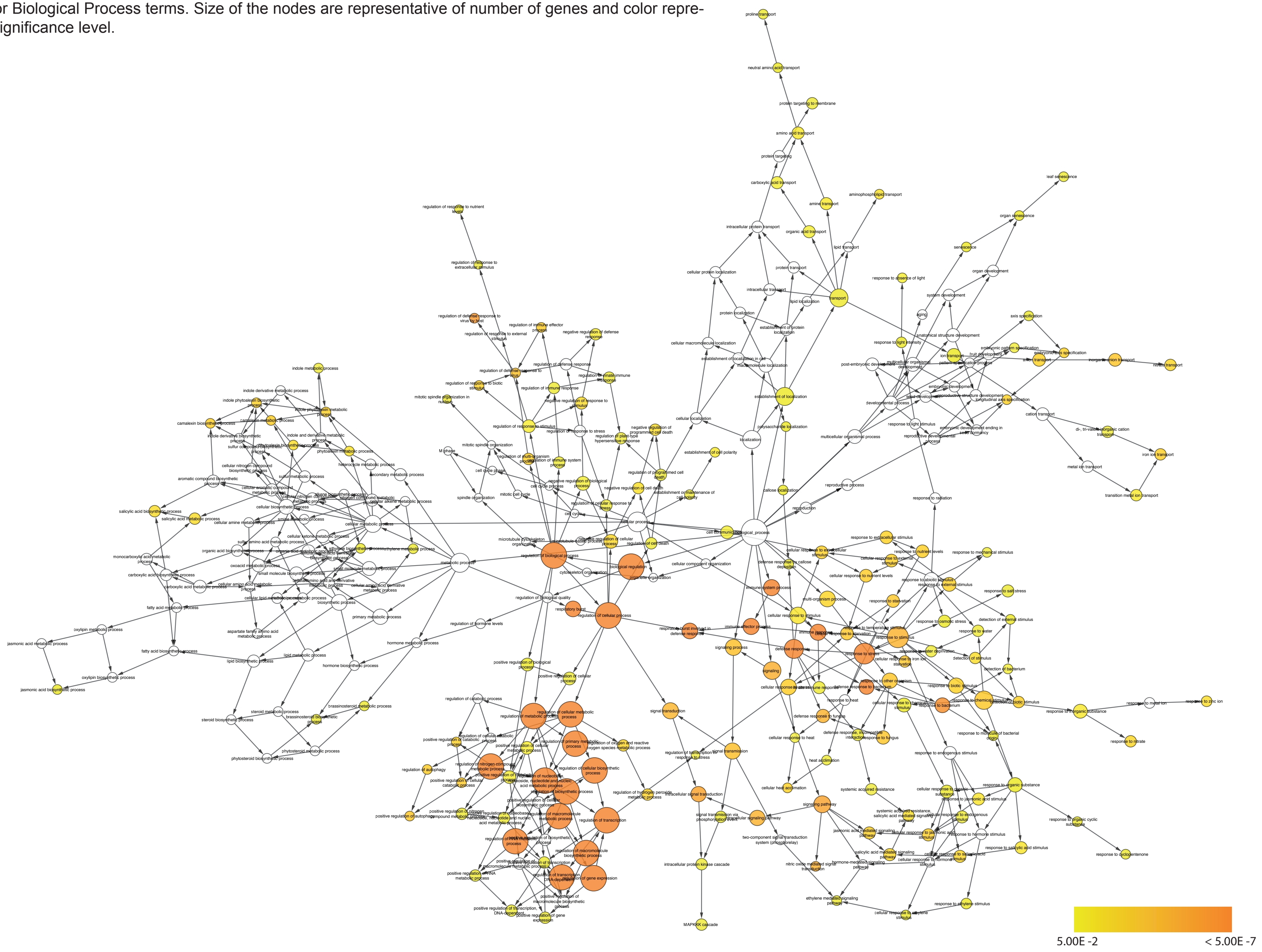

Supplementary Figure S5. Gene ontology enrichment analysis term relations are shown for WRKY genes in mulberry. A) Molecular Function and B) Cellular Components. Size of nodes shows the number of components enriched. Color represents the significance of the term (See legend).

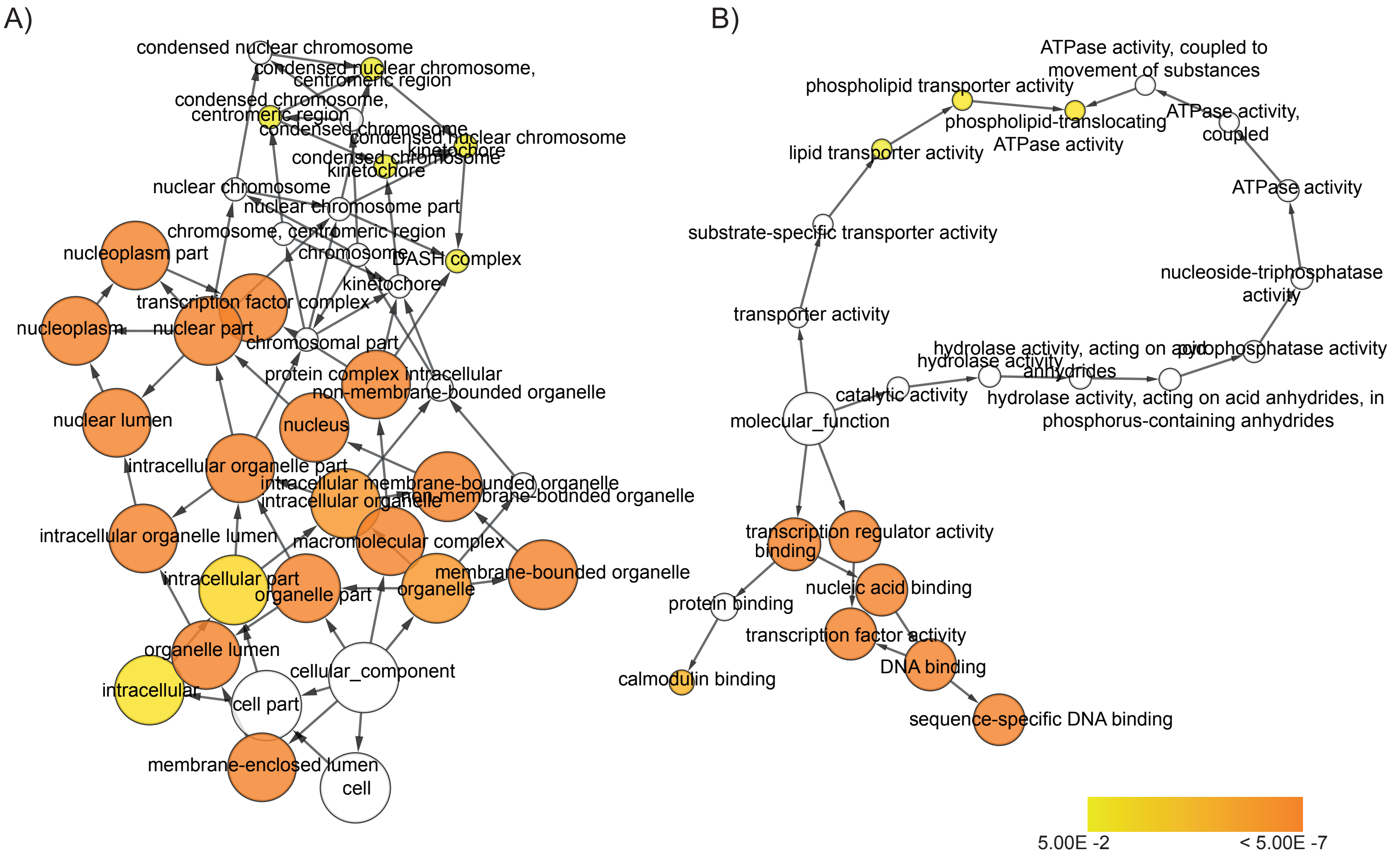

Supplementary Table S1. Structural features of WRKY genes in mulberry.

Gene ID	Group	Gene Length (bp)	mRNA Length (bp)	Exon	Intron	Alternate Spliced Forms	Mol Mass (kDa)	Isoelectric Point (pI)
Morus020954	GroupIa	3237	1518	4	3	1	41133.31	8.32
Morus014007	GroupIa	3117	1628	5	4	1	46343.46	7.67
Morus003860	GroupIa	2843	1485	5	4	1	40628.77	9.18
Morus011135	GroupIa	3013	1714	6	5	1	47601.87	7.19
Morus015723	GroupIa	2931	1656	4	3	1	44805.30	6.80
Morus009509	GroupIa	4322	1751	6	5	1	47163.34	7.31
Morus015130	GroupIa	3957	2274	5	4	1	62160.14	6.57
Morus005270	GroupIa	3430	2277	5	4	1	61572.56	6.07
Morus020026	GroupIa	2040	1071	4	3	1	29636.16	9.50
Morus009921	GroupIa	3520	1567	5	4	2	44006.20	5.58
Morus010795	GroupIIa	2224	554	2	1	1	16336.73	9.49
Morus021378	GroupIIa	2173	1166	3	2	1	33231.30	6.73
Morus006651	GroupIIa	3914	949	3	2	1	25735.51	5.10
Morus009437	GroupIIe	2382	971	3	2	1	27964.73	4.66
Morus021488	GroupIIa	2529	652	2	1	1	17939.00	10.04
Morus002784	GroupIIa	2007	1329	3	2	1	38912.52	7.02
Morus005172	GroupIIa	5171	790	3	2	1	22423.87	8.17
Morus001635	GroupIIa	4116	738	4	3	1	20173.86	7.31
Morus017459	GroupIIa	4092	622	2	1	2	18051.71	9.72
Morus019036	GroupIId	1382	940	3	2	1	25604.27	9.92
Morus003782	GroupIIb	1044	591	3	2	1	16886.32	5.80
Morus007236	GroupIId	1407	1001	3	2	1	26332.87	9.93
Morus005757	GroupIId	1794	1081	3	2	1	29501.27	10.12
Morus010126	GroupIIa	776	573	2	1	1	16780.55	9.77
Morus000558	GroupIId	1411	1124	3	2	1	30706.92	9.71
Morus019849	GroupIId	2166	1130	3	2	1	30288.45	10.23
Morus008323	GroupIIb	4088	646	3	2	1	18289.93	7.63
Morus025955	GroupIId	3205	1540	3	2	1	42117.90	6.29
Morus014218	GroupIId	1524	1316	3	2	1	37309.02	5.98
Morus014181	GroupIIc	2434	1898	6	5	1	51422.77	6.24
Morus008065	GroupIIc	3364	1794	6	5	1	48576.11	6.51
Morus012914	GroupIId	1403	1108	3	2	1	30203.47	6.79
Morus001997	GroupIIc	2704	1644	4	3	1	45345.03	6.51
Morus024154	GroupIIc	28304	2357	16	15	6	64914.08	4.99
Morus011928	GroupIIc	2703	1840	5	4	1	50655.57	7.46
Morus021097	GroupIIc	4680	1852	6	5	1	52330.03	6.47
Morus013515	GroupIIc	1534	958	5	4	1	27401.85	8.79
Morus021355	GroupIId	2134	793	3	2	1	21053.61	5.21
Morus009739	GroupIId	1244	824	3	2	1	24187.02	5.81
Morus011458	GroupIII	1956	1243	3	2	1	34820.01	6.42
Morus013216	GroupIIc	1708	879	4	3	1	24227.58	8.90
Morus006704	GroupIId	1160	1013	2	1	1	29421.62	6.15
Morus007627	GroupIIc	4018	1977	5	4	1	55449.14	6.90

Morus013217	GroupIIc	2129	1013	5	4	1	28781.68	7.83
Morus012050	GroupIII	1431	1068	3	2	1	30203.69	5.08
Morus017596	GroupIII	2584	1117	3	2	1	31742.09	5.16
Morus011258	GroupIII	2960	1062	3	2	1	29678.33	5.97
Morus000931	GroupIId	1320	915	3	2	1	24969.37	5.45
Morus011259	GroupIII	26142	1105	4	3	5	31709.59	4.94
Morus013971	GroupIII	1853	1010	3	2	1	29407.82	6.56
Morus004213	GroupIII	2250	1019	3	2	1	28532.12	6.57
Morus013969	GroupIII	2248	1019	3	2	1	28445.16	6.70
Morus013973	GroupIII	1959	1016	3	2	1	29520.66	5.78
Morus010632	GroupIVb	1757	1292	3	2	1	36526.98	6.72

Supplementary Table S2: Pfam domains distribution and scanprosite results of mulberry WRKY genes.

Groups	ID	PF08947.5	PF09692.5	PF04156.9	PF12496.3	PF03101.10	PF02183.13	PF02401.13	PF01805.15	PF15233.1	PF09371.5	PF03106.10	PF01213.14	PF14916.1	PF04977.10	PF05344.6	PF06005.7	PF00170.16	PF07716.10	PF09674.5
GroupIa	Morus020954.p1	-	-	-	-	2	-	-	-	-	-	2	-	-	-	-	-	-	-	-
GroupIa	Morus014007.p1	-	-	-	-	2	-	-	-	-	-	2	-	-	-	-	-	-	-	-
GroupIa	Morus003860.p1	-	-	-	-	2	-	-	-	-	-	2	-	-	-	-	-	-	-	-
GroupIa	Morus015723.p1	-	-	-	-	2	-	-	-	-	-	2	-	-	-	-	-	-	-	-
GroupIa	Morus015130.p1	-	-	-	-	2	-	-	-	-	-	2	-	-	-	-	-	-	-	-
GroupIa	Morus005270.p1	-	-	-	-	2	-	-	-	-	-	2	-	-	-	-	-	-	-	-
GroupIa	Morus011135.p1	-	-	-	-	3	-	-	-	-	-	2	-	-	-	-	-	-	-	-
GroupIa	Morus020026.p1	-	-	-	-	3	-	-	-	-	-	3	-	-	-	-	-	-	-	-
GroupIa	Morus009509.p1	-	-	-	-	-	-	-	-	-	-	2	-	-	-	-	-	-	-	-
GroupIa	Morus009921.p1	-	-	-	-	-	-	-	-	-	-	3	-	-	-	-	-	-	-	-
GroupIIa	Morus021378.p1	-	-	-	-	-	-	-	2	-	-	2	-	-	-	-	-	-	-	-
GroupIIa	Morus002784.p1	-	-	-	-	-	-	-	-	-	-	1	-	-	-	-	-	-	-	-
GroupIIa	Morus010795.p1	-	-	-	-	-	-	-	-	-	-	1	-	-	-	-	-	-	-	-
GroupIIa	Morus006651.p1	-	-	-	-	-	-	-	-	-	-	2	-	-	-	-	-	-	-	-
GroupIIa	Morus021488.p1	-	-	-	-	-	-	-	-	-	-	1	-	-	-	-	-	-	-	-
GroupIIa	Morus005172.p1	-	-	-	-	-	-	-	-	-	-	1	-	-	-	-	-	-	-	-
GroupIIa	Morus001635.p1	-	-	-	-	-	-	-	-	-	-	2	-	-	-	-	-	-	-	-
GroupIIa	Morus017459.p1	-	-	-	-	-	-	-	-	-	-	2	-	-	-	-	-	-	-	-
GroupIIa	Morus010126.p1	-	-	-	-	-	-	-	-	-	-	2	-	-	-	-	-	-	-	-
GroupIIb	Morus003782.p1	-	-	-	-	1	-	-	-	-	-	1	-	-	-	-	-	-	-	-
GroupIIb	Morus008323.p1	-	-	-	-	2	-	-	-	-	-	1	-	-	-	-	-	-	-	-
GroupIIc	Morus013216.p1	-	-	-	-	-	1	-	-	-	-	2	-	1	-	-	-	2	-	-
GroupIIc	Morus014181.p1	-	-	-	-	-	-	-	-	1	-	1	-	-	-	-	-	-	1	-
GroupIIc	Morus011928.p1	-	-	1	-	-	-	-	-	-	-	1	-	-	2	-	2	-	-	-
GroupIIc	Morus013515.p1	-	-	-	-	-	-	-	-	-	-	1	-	-	-	-	-	-	-	1
GroupIIc	Morus008065.p1	-	-	1	-	-	-	-	-	1	-	1	-	-	-	-	-	-	-	-
GroupIIc	Morus024154.p1	-	-	-	-	-	-	2	-	-	-	1	-	-	-	-	-	-	-	-
GroupIIc	Morus001997.p1	-	-	-	-	-	-	-	-	-	-	1	-	-	-	-	-	-	-	-
GroupIIc	Morus021097.p1	-	-	-	-	-	-	-	-	-	-	1	-	-	-	-	-	-	-	-
GroupIIc	Morus007627.p1	-	-	-	-	-	-	-	-	-	-	3	-	-	-	-	-	-	-	-
GroupIIc	Morus013217.p1	-	-	-	-	-	-	-	-	-	-	1	-	-	-	-	-	-	-	-
GroupIId	Morus000558.p1	-	1	-	-	-	-	-	-	-	-	2	-	-	-	-	-	-	-	-
GroupIId	Morus009739.p1	-	-	-	-	2	-	-	-	-	-	1	-	-	-	-	-	-	-	-
GroupIId	Morus000931.p1	-	-	-	-	2	-	-	-	-	-	1	-	-	-	-	-	-	-	-
GroupIId	Morus005757.p1	-	-	-	-	-	-	-	-	-	-	2	-	-	-	-	-	-	-	-
GroupIId	Morus007236.p1	-	-	-	-	-	-	-	-	-	-	2	1	-	-	-	-	-	-	-
GroupIId	Morus019849.p1	-	-	-	-	-	-	-	-	-	-	2	-	-	-	2	-	-	-	-
GroupIId	Morus019036.p1	-	-	-	-	-	-	-	-	-	-	1	-	-	-	-	-	-	-	-
GroupIId	Morus012914.p1	-	-	-	-	-	-	-	-	-	-	1	-	-	-	-	-	-	-	-
GroupIId	Morus006704.p1	-	-	-	-	-	-	-	-	-	2	3	-	-	-	-	-	-	-	-
GroupIId	Morus025955.p1	-	-	-	-	-	-	-	-	-	-	1	-	-	-	-	-	-	-	-
GroupIId	Morus014218.p1	-	-	-	-	-	-	-	-	-	-	1	-	-	-	-	-	-	-	-
GroupIId	Morus021355.p1	-	-	-	-	-	-	-	-	-	-	1	-	-	-	-	-	-	-	-
GroupIIe	Morus009437.p1	-	-	-	-	-	-	-	-	-	-	1	-	-	-	-	-	-	-	-
GroupIII	Morus011259.p1	1	-	-	-	-	-	-	-	-	-	2	-	-	-	-	-	-	-	-
GroupIII	Morus017596.p1	-	-	-	2	-	-	-	-	-	-	1	-	-	-	-	-	-	-	-
GroupIII	Morus004213.p1	-	-	-	-	-	-	-	-	-	-	1	-	-	-	-	-	-	-	-
GroupIII	Morus013969.p1	-	-	-	-	-	-	-	-	-	-	1	-	-	-	-	-	-	-	-
GroupIII	Morus013973.p1	-	-	-	-	-	-	-	-	-	-	1	-	-	-	-	-	-	-	-
GroupIII	Morus011258.p1	-	-	-	-	-	-	-	-	-	-	2	-	-	-	-	-	-	-	-
GroupIII	Morus013971.p1	-	-	-	-	-	-	-	-	-	-	2	-	-	-	-	-	-	-	-
GroupIII	Morus011458.p1	-	-	-	-	-	-	-	-	-	-	1	-	-	-	-	-	-	-	-
GroupIII	Morus012050.p1	-	-	-	-	-	-	-	-	-	-	1	-	-	-	-	-	-	-	-
GroupIVb	Morus010632.p1	-	-	-	-	-	-	-	-	-	-	1	-	-	-	-	-	-	-	-

Groups	ID	PF13334.1	PF14975.1	PF06743.10	PF04500.11	PF14812.1	PF06459.7	PF04684.8	PF04277.8	PF04606.7	PF01496.14	PF05812.7	PF13445.1	PF01433.15	PF13759.1	PF04696.8	PF10533.4	PF12709.2	PF05148.10	PF13408.1
GroupIa	Morus020954.p1	-	-	-	-	-	-	-	-	-	-	-	-	-	-	-	-	-	-	-
GroupIa	Morus014007.p1	-	-	-	3	-	-	-	-	2	-	-	-	-	-	-	-	-	-	-
GroupIa	Morus003860.p1	-	-	-	-	-	-	-	-	-	-	-	-	-	-	-	-	-	-	-
GroupIa	Morus015723.p1	-	-	-	-	-	-	-	-	-	-	-	-	-	-	-	-	-	-	-
GroupIa	Morus015130.p1	-	-	-	-	-	-	-	-	-	-	-	-	-	-	-	-	-	-	-
GroupIa	Morus005270.p1	-	-	-	-	-	-	-	-	-	-	-	-	-	-	-	-	-	-	-
GroupIa	Morus011135.p1	-	3	-	-	-	-	-	-	-	-	-	-	-	-	-	-	-	-	-
GroupIa	Morus020026.p1	-	-	-	-	-	-	-	-	2	-	-	-	-	-	-	-	-	-	-
GroupIa	Morus009509.p1	-	-	-	2	-	-	-	-	2	-	-	-	-	-	-	-	-	-	-
GroupIa	Morus009921.p1	-	-	-	-	-	-	-	-	-	-	-	-	-	-	-	-	-	-	-
GroupIIa	Morus021378.p1	-	-	-	-	1	1	-	-	-	-	-	-	-	-	-	-	-	-	-
GroupIIa	Morus002784.p1	-	-	-	-	-	1	-	-	-	-	-	-	-	-	-	-	-	-	-
GroupIIa	Morus010795.p1	-	-	-	-	-	-	-	-	-	-	-	-	-	-	-	-	-	-	-
GroupIIa	Morus006651.p1	-	-	-	-	-	-	-	-	-	-	-	-	-	-	-	-	-	-	-
GroupIIa	Morus021488.p1	-	-	-	-	-	-	-	-	-	-	-	-	-	-	-	-	-	-	-
GroupIIa	Morus005172.p1	-	-	-	-	-	-	-	-	-	-	-	-	-	-	-	-	-	-	-
GroupIIa	Morus001635.p1	-	-	-	-	-	-	-	-	-	-	-	-	-	-	-	-	-	-	-
GroupIIa	Morus017459.p1	-	-	-	-	-	-	-	-	-	-	-	-	-	-	-	-	-	-	-
GroupIIa	Morus010126.p1	-	-	-	-	-	-	-	-	-	-	-	-	-	-	-	-	-	-	-
GroupIIb	Morus003782.p1	-	-	-	-	-	-	-	-	-	-	-	-	-	-	-	-	-	-	-
GroupIIb	Morus008323.p1	-	-	-	-	-	-	-	-	-	-	-	-	-	-	-	-	-	-	-
GroupIIc	Morus013216.p1	-	-	-	-	-	-	-	-	-	-	-	-	-	2	-	-	2	-	-
GroupIIc	Morus014181.p1	-	-	-	-	-	-	-	-	-	1	-	-	-	-	-	-	-	-	-
GroupIIc	Morus011928.p1	1	-	-	-	-	-	-	-	-	1	1	-	-	-	-	-	-	-	-
GroupIIc	Morus013515.p1	-	-	-	-	-	-	-	2	-	-	-	-	-	-	-	-	-	-	-
GroupIIc	Morus008065.p1	-	-	-	-	-	-	-	-	-	-	-	-	-	-	-	-	-	-	-
GroupIIc	Morus024154.p1	-	-	-	-	-	-	-	-	-	-	-	-	-	-	5	-	-	-	-
GroupIIc	Morus001997.p1	-	-	-	-	-	-	-	-	-	-	-	-	-	-	-	-	-	-	-
GroupIIc	Morus021097.p1	-	-	-	-	-	-	-	-	-	-	-	-	-	-	-	-	-	-	-
GroupIIc	Morus007627.p1	-	-	-	-	-	-	-	-	-	-	-	-	-	-	-	-	-	-	-
GroupIIc	Morus013217.p1	-	-	-	-	-	-	-	-	-	-	-	-	-	-	-	-	-	-	-
GroupIId	Morus000558.p1	-	-	-	-	-	-	-	-	-	-	-	-	-	-	-	1	-	-	-
GroupIId	Morus009739.p1	-	-	-	-	-	-	-	-	-	-	-	-	-	-	-	-	-	-	-
GroupIId	Morus000931.p1	-	-	-	-	-	-	-	-	-	-	-	-	-	-	-	-	-	-	-
GroupIId	Morus005757.p1	-	-	-	-	-	-	1	-	-	-	-	-	-	-	-	1	-	-	-
GroupIId	Morus007236.p1	-	-	-	-	-	-	-	-	-	-	-	-	-	-	-	1	-	-	-
GroupIId	Morus019849.p1	-	-	-	-	-	-	-	-	-	-	-	-	-	-	-	1	-	-	-
GroupIId	Morus019036.p1	-	-	-	-	-	-	-	-	-	-	-	-	-	-	-	1	-	-	-
GroupIId	Morus012914.p1	-	-	-	-	-	-	-	-	-	-	-	-	-	-	-	1	-	-	-
GroupIId	Morus006704.p1	-	-	-	-	-	-	-	-	-	-	-	-	-	-	-	-	-	-	-
GroupIId	Morus025955.p1	-	-	-	-	-	-	-	-	-	-	-	-	-	-	-	-	-	-	-
GroupIId	Morus014218.p1	-	-	-	-	-	-	-	-	-	-	-	-	-	-	-	-	-	-	-
GroupIId	Morus021355.p1	-	-	-	-	-	-	-	-	-	-	-	-	-	-	-	-	-	-	-
GroupIIe	Morus009437.p1	-	-	-	-	-	-	-	-	1	-	-	-	-	-	-	-	-	-	-
GroupIII	Morus011259.p1	-	-	-	-	-	-	-	-	-	-	-	-	-	-	-	-	-	-	-
GroupIII	Morus017596.p1	-	-	-	-	-	-	-	-	-	-	-	-	-	-	-	-	-	-	-
GroupIII	Morus004213.p1	-	-	2	-	-	-	-	-	-	-	-	1	-	-	-	-	-	-	2
GroupIII	Morus013969.p1	-	-	2	-	-	-	-	-	-	-	-	1	-	-	-	-	-	-	2
GroupIII	Morus013973.p1	-	-	-	2	-	-	-	-	-	-	-	1	-	-	-	-	-	-	2
GroupIII	Morus011258.p1	-	-	-	-	-	-	-	-	-	-	-	-	-	-	-	-	-	2	-
GroupIII	Morus013971.p1	-	-	-	-	-	-	-	-	-	-	-	-	1	-	-	-	-	-	3
GroupIII	Morus011458.p1	-	-	-	-	-	-	-	-	-	-	-	-	-	-	-	-	-	-	-
GroupIII	Morus012050.p1	-	-	-	-	-	-	-	-	-	-	-	-	-	-	-	-	-	-	-
GroupIVb	Morus010632.p1	-	-	-	-	-	-	-	-	-	-	-	-	-	-	-	-	-	-	-

ScanProsite Results

MorusDB ID	Start	End	Domain	Domain Sequence
Morus020954	238	302	PS50811|WRKY	ATDKPADDGYNWRKYGQKQVKGSEFPRSYYKCTHLNCPVKKKVER-SPDGQITEIIYKGQHNHEKP
Morus020954	414	479	PS50811|WRKY	SEVDLLDDGYRWRKYGQKVVKGNPHPRSYYKCTNAGCNVRKHVERASTDPKAVITTYEGKHNHDVP
Morus014007	190	254	PS50811|WRKY	VREQKSDDGYNWRKYGQKQVKGSENPRSYYKCTNPNCPMKKKVER-SLDGQITEIVYKGSHNHPKP
Morus014007	350	415	PS50811|WRKY	SEIDILDDGYRWRKYGQKVVKGNPNPRSYYKCTSVGCPVRKHVERASHDTRAVITTYEGKHNHDVP
Morus003860	198	255	PS50811|WRKY	-------DGYNWRKYGQKQVKGSEFPRSYYKCTHPNCPVKKKVER-SLDGQIAEIVYKGEHNHSKP
Morus003860	382	447	PS50811|WRKY	SDPEISGDGFRWRKYGQKVVKGSPYPRSYYRCTSLKCNVRKHVERASDDPRAFITTYEGRHNHDMP
Morus011135	202	266	PS50811|WRKY	SLSRRSDDGYNWRKYGQKQVKGSENPRSYYKCTYPNCPTKKKVERA-LDGQITEIVYKGSHNHPKP
Morus011135	372	437	PS50811|WRKY	SDIDILDDGYRWRKYGQKVVKGNPNPRSYYKCTNPGCPVRKHVERASHDLRAVITTYEGKHNHDVP
Morus015723	240	304	PS50811|WRKY	SFDKPNDDGYNWRKYGQKQVKGSEFPRSYYKCTHPNCPVKKKVERSLEGQVTEII-YKGQHNHQRP
Morus015723	420	485	PS50811|WRKY	SEVDLLDDGYRWRKYGQKVVKGNPYPRSYYKCTTTGCNVRKHVERASSDSKAVITTYEGKHNHDVP
Morus009509	216	274	PS50811|WRKY	------DDGYNWRKYGQKHVKGSEFPRSYYKCTHPNCEVKKLFER-SHDGQITEIIYKGTHDHPKP
Morus009509	383	448	PS50811|WRKY	SEVDILDDGYRWRKYGQKVVRGNPNPRSYYKCTNAGCPVRKHVERASHDPKAVITTYEGKHNHDVP
Morus015130	258	316	PS50811|WRKY	------EDGYNWRKYGQKQVKGSEYPRSYYKCTHTDCQVKKKVER-SHDGQITEIIYKGSHNHAKP
Morus015130	472	537	PS50811|WRKY	SDVDILDDGYRWRKYGQKVVKGNPNPRSYYKCTSAGCSVRKHVERASHNLKFVITTYEGKHNHEVP
Morus005270	315	379	PS50811|WRKY	GGGTVSEDGYNWRKYGQKQVKGSEYPRSYYKCTHLNCQVKKKVER-SHEGHITEIIYKGSHNHPKP
Morus005270	527	592	PS50811|WRKY	SEVDILDDGYRWRKYGQKVVKGNPNPRSYYKCTNAGCTVRKHVERASHDLKSVITTYEGKHNHDVP
Morus020026	30	94	PS50811|WRKY	IREKVAQDGYNWRKYGQKLVKGNEYVRSYYRCTHPNCQVKKQLE-CSHDRQIVDIVYFGHHDHPKR
Morus020026	205	270	PS50811|WRKY	SEVDIVNDGHRWRKYGQKIVKGSPHPRSYYRCSNSGCPVKKHVERASHDAKVVITTYEGEHNHGMP
Morus009921	192	250	PS50811|WRKY	------SDGYNWRKYGQKQVKSPEGSRSYYKCTHSECYAKK-IECCDHSGSVTEIVYKSQHSHDPP
Morus009921	367	432	PS50811|WRKY	GDVGISGDGYRWRKYGQKMVKGNPHPRNYYRCTSAGCPVRKHIETAVDNMNAVIITYKGIHDHDMP
Morus010795	96	161	PS50811|WRKY	SQVDILDDGYRWRKYGQKAVKNNKFPRSYYRCTHQGCNVKKQVQRLTKDEGVVVTTYEGMHTHPIE
Morus021378	220	285	PS50811|WRKY	SEIDHLEDGYRWRKYGQKAVKNSPYPRSYYRCTTQKCTVKKRVERSFQDPSIVITTYEGQHNHHCP
Morus006651	155	220	PS50811|WRKY	SEVDHLEDGYRWRKYGQKAVKNSPFPRSYYRCTNSKCIVKKRVERSSEDPTIVITTYEGQHCHHTV
Morus009437	125	187	PS50811|WRKY	CGNGMADDGYKWRKYGQKSIKNSPNPRSYYRCTNPRCSAKKQVERSSDDPDTLIITYEGLHLH---
Morus021488	128	193	PS50811|WRKY	SADDILDDGYRWRKYGQKAVKNSSYPRSYYRCTHHTCNVKKQVQRLSKDTSIVVTTYEGIHNHPCE
Morus002784	216	281	PS50811|WRKY	SDVDHLDDGYRWRKYGQKAVKNNPYPRSYYRCTSGRCGVKKRVERSSDDATIVVTTYEGQHTHPCP
Morus005172	175	240	PS50811|WRKY	SEVDVLDDGYKWRKYGQKVVKNTQHPRSYYRCTQDNCRVKKRVERLAEDPRMVITTYEGRHVHSPS
Morus001635	161	226	PS50811|WRKY	SDVDVLDDGYKWRKYGQKVVKNSLHPRSYYRCTHNNCRVKKRVERLSEDCRMVITTYEGRHNHSPS
Morus017459	118	183	PS50811|WRKY	SQVDILDDGYRWRKYGQKAVKNNKFPRSYYRCTHQGCNVKKQVQRLTKDEGVVVTTYEGMHSHAIE
Morus019036	227	293	PS50811|WRKY	KSADIPPDDYSWRKYGQKPIKGSPYPRSYYKCsSVKGCPARKHVERCLDDPTMLVVTYEGDHNHSKI
Morus003782	126	191	PS50811|WRKY	SEAEILDDGFKWRKYGKKMVKNSPNPRNYYRCSVEGCPVKKRVERDREDPRYVITTYEGVHNHQSS
Morus007236	248	314	PS50811|WRKY	KMADIPPDDYSWRKYGQKPIKGSPHPRGYYKCsSVRGCPARKHVERALDDPSMLVVTYEGEHNHSLS
Morus005757	279	345	PS50811|WRKY	KLADIPPDDYSWRKYGQKPIKGSPHPRGYYKCsSMRGCPARKHVERCLEDPSMLIVTYEGEHNHPRI
Morus010126	104	169	PS50811|WRKY	SQVDVLDDGYRWRKYGQKTVKNSKFPRSYYKCTYQGCNVKKQIQRHCKDEGIVVTTYEGMHTHPLE
Morus000558	286	352	PS50811|WRKY	KMADIPPDEYSWRKYGQKPIKGSPHPRGYYKCsSLRGCPARKHVERALDDPAMLIVTYEGDHNHTQS
Morus019849	276	342	PS50811|WRKY	KIADIPQDEYSWRKYGQKPIKGSPYPRGYYKCsTMRGCPARKHVERAPDDPAMLIVTYEGEHRHAHA
Morus008323	117	182	PS50811|WRKY	TEQDVLDDGFRWRKYGKKSVKNSPNPRNYYKCSSGGCNVKKRVERDRDDSSYVITTYEGVHNHESP
Morus025955	255	321	PS50811|WRKY	SGEVVPSDLWAWRKYGQKPIKGSPYPRGYYRCsSSKGCSARKQVERSRTDPNMLVVTYTSEHNHPWP
Morus014218	240	306	PS50811|WRKY	TAENLNSDLWAWRKYGQKPIKGSPYPRNYYRCsSSKGCAARKQVERSNVDPDLFIVTYTGEHTHPRP
Morus014181	340	406	PS50811|WRKY	SEAPMITDGCQWRKYGQKMAKGNPCPRAYYRCtMAVGCPVRKQVQRCAEDRTILITTYEGNHNHPLP
Morus008065	322	388	PS50811|WRKY	SEASMLSDGCQWRKYGQKMAKGNPCPRAYYRCtMATGCPVRKQVQRCAEDQTILTTTYEGHHNHPLP
Morus012914	171	237	PS50811|WRKY	PAESLSSDIWAWRKYGQKPIKGSPYPRGYYRCsSSKGCLARKQVERNRSDPGMFIVTYTAEHNHPAP
Morus001997	232	298	PS50811|WRKY	CDTPTMNDGCQWRKYGQKIAKGNPCPRAYYRCtVAPSCPVRKQVQRCAEDMSILTTTYEGTHNHPLP
Morus024154	255	321	PS50811|WRKY	CEAATLNDGCQWRKYGQKIAKGNPCPRAYYRCtVAPGCPVRKQVQRCLEDMSILITTYEGNHNHPLP
Morus011928	321	387	PS50811|WRKY	SEAPMIADGCQWRKYGQKMAKGNPCPRAYYRCtMAAGCPVRKQVQRCAEEQTILVTTYEGNHNHPLP
Morus021097	300	366	PS50811|WRKY	CDAPTMNDGCQWRKYGQKIAKGNPCPRAYYRCtVSPTCPVRKQVQRCAEDMSILITTYEGTHNHPLP
Morus013515	154	220	PS50811|WRKY	DTSLIVKDGYQWRKYGQKVTRDNPSPRAYFKCsFAPSCPVKKKVQRSVEDQSILVATYEGEHNHPQP
Morus021355	71	131	PS50811|WRKY	------SDSWAWRKYGQKPIKGSPYPRGYYRCsSSKGCPARKQVERSRVDPTTLIITYACNHNHPVP
Morus009739	56	116	PS50811|WRKY	------SDFWSWRKYGQKPIKGSPYPRGYYRCsTSKGCSAKKQVERCKTDDSLLIITYTSSHNHPGP
Morus011458	132	195	PS50811|WRKY	GLEGPLDDGFSWRKYGQKDILGAKYPRGYYRCthrNVQGCLATKQVQRSDEDPNIFEITYRGRH-----
Morus013216	98	157	PS50811|WRKY	-------DGCQWRKYGQKVTKDNPFPRAYFRCsMAPGCPVKKKVQRSMEDKSILVATYEGQHNHDAR
Morus006704	129	195	PS50811|WRKY	KEDGLCADMWAWRKYGQKPIKGSPYPRSYYRCsSSKGCSARKQVERSPSNPEIFVITYTAEHSHTHP
Morus007627	262	328	PS50811|WRKY	CDTPTMNDGCQWRKYGQKIAKGNPCPRAYYRCtVAPSCPVRKQVQRYADDISILITTYEGTHNHPLP
Morus013217	175	234	PS50811|WRKY	-------DGYQWRKYGQKVTRDNPSPRAYFKCsFAPCCPVKKKVQRSAEDPTLLVVTYEGEHSHKNP
Morus012050	122	185	PS50811|WRKY	GLEGPQDDGYSWRKYGQKDILGAKYPRSYYRCtfrNTQSCWATKQVQRSDEDPYLFEITYRGTH-----
Morus017596	115	178	PS50811|WRKY	GPEGPLEDGYSWRKYGQKDILGANHPRGYYRCtrrNAQGCLATKQVQRSDQDSSLFEVTYRGRH-----
Morus011258	148	210	PS50811|WRKY	------DDGYTWRKYGQKEIMGSKYPRGYYRCthqKLYDCPAKKQVQRLDHDPSTFEVIYHSHHTCHMS
Morus000931	116	178	PS50811|WRKY	SGSTPPSDSWAWRKYGQKPIKGSPYPR----CsTSKGCPARKQVERSRVDPTMLVVTYSAEHNHPWP
Morus011259	131	189	PS50811|WRKY	-----LEDGQAWRKYGQKDILNAQYPRAYFRCtrkYDQGCRATKQVQQIQDNPKLYKITYIGQH-----
Morus013971	133	196	PS50811|WRKY	ETSTLMDDGHAWRKYGQKMILKGKHPRHYYRCthkNDQGCQATKQVQKIQDNPPIFRTKYYGHH-----
Morus004213	133	196	PS50811|WRKY	ETSTSIDDGHAWRKYGQKMILKAKHPRHYYRCtnkYDQGCQATKQFQKIQDNPPIFRTTYCGHH-----
Morus013969	133	196	PS50811|WRKY	ETSTSIDDGHAWRKYGQKMILKAKHPRHYYRCtnkYDQGCQATKQFQKIQDNPPIFRTTYCGHH-----
Morus013973	131	194	PS50811|WRKY	ETSTLTDDGHAWRKYGQKMIIKGKHPRHYYRCtnkYDQGCQATKQVQKIQESPPIFRTTYCGHH-----
Morus010632	207	276	PS50811|WRKY	SEVDHLEDGYRWRKYGQKAVKNSPYPSLSHSHhfmlCAQKLLSLHHSKMRRQETRREIFSGSVHSDNDVR

Supplementary Table S3: Distribution of cis elements in 2K upstream regions of mulberry WRKY genes.

Gene wise distribution of *cis* elements in 2K upstream regions

Gene ID	ABRE	ARF Binding Site	ASF-1 binding site	AUXRE	Core of GCC Box	DRE1 Core	DREB Binding Site	Drought Responsive	GT-1 motif	LTRE
Morus009739	1				1				1	
Morus012914		5					1		8	4
Morus013217		1	3		1		2	1	1	3
Morus011258		2	1		1				6	
Morus009509			1						1	
Morus005757			1						13	
Morus002784			1				2		9	
Morus009921			1	2			2		13	3
Morus013971										
Morus020954	2	1			1		1		1	2
Morus014007	2	1			1		1		7	1
Morus010632	2	1	1				2		11	3
Morus000931	1		2				2		8	1
Morus015723	2		1						4	1
Morus015130	1	1	4				2		9	1
Morus000558	1	1	5						7	
Morus008065	2		4						1	
Morus001997	1	1	2				1		4	3
Morus008323	1				1				1	
Morus019849	1		1				1		14	3
Morus005172		1							7	
Morus013216		2	2				1		8	1
Morus021378		1	4						6	
Morus010795		2	1		2				9	1
Morus003782		1	4		1				12	3
Morus013515		3					1		1	2
Morus005270		3	1						17	
Morus013973		1							3	
Morus019036		5					1		8	4
Morus021097		1	3		1		1		6	1
Morus025955		1			1				12	3
Morus011135		1			1				8	1
Morus021355		1	1	1					6	
Morus012050		1					1		8	1
Morus007627			2						11	4
Morus001635			1						1	
Morus010126			3		4		1		5	1
Morus017459			1		1				5	
Morus007236		1	3		1		2	1	1	3
Morus004213			6				2		8	3
Morus011259			4				1		7	
Morus009437			1						1	
Morus006704			1				1		6	2
Morus011928			4				5		16	3
Morus017596			4						13	
Morus011458			1			2			9	
Morus014181			7				2		8	4
Morus003860			1						12	
Morus006651		2	1		1				6	
Morus020026					1				9	4
Morus014218									9	2
Morus024154									3	1
Morus021488									3	1

Gene ID	Motif for drought, low temp or high-salt stress	T/G-box for Jasmonate Induction	TCA-1 binding site; SA Inducible	W Box recognized by SA induced WRKYs
Morus009739		1		6
Morus012914				2
Morus013217	1	1		3
Morus011258				6
Morus009509				2
Morus005757				4
Morus002784		1		2
Morus009921		1		4
Morus013971				
Morus020954				1
Morus014007				7
Morus010632		1		8
Morus000931				4
Morus015723		2		2
Morus015130				4
Morus000558		2		11
Morus008065		1		1
Morus001997				6
Morus008323		1		6
Morus019849				7
Morus005172				3
Morus013216				4
Morus021378				6
Morus010795				
Morus003782				7
Morus013515		2		5
Morus005270		3		1
Morus013973				3
Morus019036				2
Morus021097				11
Morus025955				2
Morus011135		2		9
Morus021355				1
Morus012050				3
Morus007627				5
Morus001635				2
Morus010126		1		4
Morus017459		2		2
Morus007236	1	1		3
Morus004213				3
Morus011259			1	4
Morus009437		2		4
Morus006704		2		11
Morus011928		3		4
Morus017596				2
Morus011458				1
Morus014181		1		8
Morus003860		1		7
Morus006651				6
Morus020026		1		3
Morus014218				3
Morus024154		1		7
Morus021488		6		5

Supplementary Table S4. Gene ontology enrichment analysis result for biological process terms.

GO-ID	P-value (Corrected)	Description
GO:0045449	9.6389E-42	regulation of transcription
GO:0006355	2.5239E-40	regulation of transcription, DNA-dependent
GO:0051252	4.8534E-39	regulation of RNA metabolic process
GO:0019219	2.0589E-38	regulation of nucleobase, nucleoside, nucleotide and nucleic acid metabolic process
GO:0051171	2.4936E-38	regulation of nitrogen compound metabolic process
GO:0010556	1.9065E-37	regulation of macromolecule biosynthetic process
GO:0010468	6.3189E-37	regulation of gene expression
GO:0031326	8.7728E-37	regulation of cellular biosynthetic process
GO:0009889	8.9243E-37	regulation of biosynthetic process
GO:0060255	6.2483E-35	regulation of macromolecule metabolic process
GO:0080090	1.2179E-33	regulation of primary metabolic process
GO:0031323	1.1327E-31	regulation of cellular metabolic process
GO:0019222	5.3126E-31	regulation of metabolic process
GO:0050794	5.4394E-27	regulation of cellular process
GO:0050789	4.5079E-25	regulation of biological process
GO:0065007	4.0432E-22	biological regulation
GO:0045730	6.6373E-18	respiratory burst
GO:0002679	6.6373E-18	respiratory burst involved in defense response
GO:0002252	1.6304E-14	immune effector process
GO:0006952	4.016E-11	defense response
GO:0006955	2.7934E-08	immune response
GO:0002376	3.762E-08	immune system process
GO:0042742	2.3723E-07	defense response to bacterium
GO:0006950	3.0269E-07	response to stress
GO:0009617	1.7356E-06	response to bacterium
GO:0050691	6.6826E-06	regulation of defense response to virus by host
GO:0035556	0.000015036	intracellular signal transduction
GO:0023033	0.000016328	signaling pathway
GO:0023052	0.000047068	signaling
GO:0007263	0.000057847	nitric oxide mediated signal transduction
GO:0023034	0.000060132	intracellular signaling pathway
GO:0050832	0.000060814	defense response to fungus
GO:0050896	0.000070998	response to stimulus
GO:0007165	0.000093498	signal transduction
GO:0023060	0.00019877	signal transmission
GO:0023046	0.00019877	signaling process
GO:0050688	0.0003087	regulation of defense response to virus
GO:0015698	0.00033682	inorganic anion transport
GO:0051707	0.00036387	response to other organism
GO:0009620	0.00036387	response to fungus
GO:0010508	0.0003838	positive regulation of autophagy
GO:0042221	0.00038986	response to chemical stimulus
GO:0010106	0.00041238	cellular response to iron ion starvation
GO:0009267	0.00041238	cellular response to starvation
GO:0042594	0.00043722	response to starvation
GO:0031331	0.00057424	positive regulation of cellular catabolic process
GO:0031669	0.00062223	cellular response to nutrient levels
GO:0002697	0.00066892	regulation of immune effector process
GO:0010506	0.00066892	regulation of autophagy
GO:0033554	0.00069532	cellular response to stress
GO:0031667	0.00073356	response to nutrient levels
GO:0052317	0.00075129	camalexin metabolic process
GO:0010120	0.00075129	camalexin biosynthetic process
GO:0006826	0.00075529	iron ion transport
GO:0009867	0.00082992	jasmonic acid mediated signaling pathway
GO:0071395	0.00082992	cellular response to jasmonic acid stimulus
GO:0009595	0.0008405	detection of biotic stimulus
GO:0031668	0.0008535	cellular response to extracellular stimulus
GO:0071496	0.0008535	cellular response to external stimulus
GO:0043900	0.00086316	regulation of multi-organism process
GO:0006820	0.00092898	anion transport
GO:0009605	0.00092898	response to external stimulus
GO:0009896	0.00092898	positive regulation of catabolic process
GO:0009942	0.00092898	longitudinal axis specification
GO:0002831	0.00092898	regulation of response to biotic stimulus
GO:0009991	0.0009398	response to extracellular stimulus

GO:0009607	0.00098903	response to biotic stimulus
GO:0070370	0.0010263	cellular heat acclimation
GO:0000578	0.0010528	embryonic axis specification
GO:0046217	0.0010528	indole phytoalexin metabolic process
GO:0009700	0.0010528	indole phytoalexin biosynthetic process
GO:0010310	0.0011895	regulation of hydrogen peroxide metabolic process
GO:0080010	0.001203	regulation of oxygen and reactive oxygen species metabolic process
GO:0051704	0.0012597	multi-organism process
GO:0009863	0.0013108	salicylic acid mediated signaling pathway
GO:0015706	0.0013416	nitrate transport
GO:0010167	0.0013416	response to nitrate
GO:0071446	0.0013416	cellular response to salicylic acid stimulus
GO:0048523	0.0014017	negative regulation of cellular process
GO:0009697	0.002065	salicylic acid biosynthetic process
GO:0048585	0.0021616	negative regulation of response to stimulus
GO:0009696	0.0022183	salicylic acid metabolic process
GO:0010043	0.0024628	response to zinc ion
GO:0051606	0.0027446	detection of stimulus
GO:0043069	0.0027446	negative regulation of programmed cell death
GO:0052315	0.0028321	phytoalexin biosynthetic process
GO:0060548	0.0029445	negative regulation of cell death
GO:0009862	0.0031886	systemic acquired resistance, salicylic acid mediated signaling pathway
GO:0052314	0.0034512	phytoalexin metabolic process
GO:0015917	0.0034512	aminophospholipid transport
GO:0051716	0.0042839	cellular response to stimulus
GO:0009753	0.0045695	response to jasmonic acid stimulus
GO:0009751	0.0045704	response to salicylic acid stimulus
GO:0010035	0.0052063	response to inorganic substance
GO:0006865	0.0053334	amino acid transport
GO:0015837	0.0060985	amine transport
GO:0030010	0.0061879	establishment of cell polarity
GO:0000041	0.0065736	transition metal ion transport
GO:0031348	0.0069446	negative regulation of defense response
GO:0048583	0.0072688	regulation of response to stimulus
GO:0031325	0.0078817	positive regulation of cellular metabolic process
GO:0009893	0.0081321	positive regulation of metabolic process
GO:0009646	0.0086774	response to absence of light
GO:0046942	0.009984	carboxylic acid transport
GO:0048519	0.010135	negative regulation of biological process
GO:0015849	0.010135	organic acid transport
GO:0007154	0.010135	cell communication
GO:0016045	0.012251	detection of bacterium
GO:0009612	0.014891	response to mechanical stimulus
GO:0009880	0.016713	embryonic pattern specification
GO:0034605	0.016713	cellular response to heat
GO:0010363	0.018527	regulation of plant-type hypersensitive response
GO:0042431	0.018598	indole metabolic process
GO:0009627	0.019602	systemic acquired resistance
GO:0043620	0.019867	regulation of transcription in response to stress
GO:0080135	0.019947	regulation of cellular response to stress
GO:0009414	0.021572	response to water deprivation
GO:0009415	0.024045	response to water
GO:0007163	0.025086	establishment or maintenance of cell polarity
GO:0015674	0.025281	di-, tri-valent inorganic cation transport
GO:0048522	0.026775	positive regulation of cellular process
GO:0043067	0.026775	regulation of programmed cell death
GO:0000165	0.027539	MAPKKK cascade
GO:0007243	0.027544	intracellular protein kinase cascade
GO:0010941	0.027933	regulation of cell death
GO:0006612	0.028325	protein targeting to membrane
GO:0045088	0.028486	regulation of innate immune response
GO:0030472	0.031081	mitotic spindle organization in nucleus
GO:0010150	0.031081	leaf senescence
GO:0052542	0.031105	defense response by callose deposition
GO:0071495	0.031294	cellular response to endogenous stimulus
GO:0050776	0.031434	regulation of immune response
GO:0045087	0.031434	innate immune response
GO:0010286	0.031468	heat acclimation

GO:0045893	0.031804	positive regulation of transcription, DNA-dependent
GO:0002682	0.032149	regulation of immune system process
GO:0051254	0.032149	positive regulation of RNA metabolic process
GO:0009651	0.033865	response to salt stress
GO:0032107	0.034584	regulation of response to nutrient levels
GO:0032104	0.034584	regulation of response to extracellular stimulus
GO:0045941	0.036761	positive regulation of transcription
GO:0010628	0.036761	positive regulation of gene expression
GO:0009692	0.036963	ethylene metabolic process
GO:0009693	0.036963	ethylene biosynthetic process
GO:0015824	0.037245	proline transport
GO:0016132	0.037322	brassinosteroid biosynthetic process
GO:0045935	0.037322	positive regulation of nucleobase, nucleoside, nucleotide and nucleic acid metabolic process
GO:0023014	0.037693	signal transmission via phosphorylation event
GO:0051173	0.037693	positive regulation of nitrogen compound metabolic process
GO:0009798	0.039456	axis specification
GO:0010583	0.039734	response to cyclopentenone
GO:0016131	0.039742	brassinosteroid metabolic process
GO:0009814	0.03987	defense response, incompatible interaction
GO:0009873	0.040029	ethylene mediated signaling pathway
GO:0006810	0.043278	transport
GO:0014070	0.043582	response to organic cyclic substance
GO:0002237	0.043853	response to molecule of bacterial origin
GO:0015804	0.043853	neutral amino acid transport
GO:0071310	0.043853	cellular response to organic substance
GO:0010149	0.043853	senescence
GO:0071369	0.043853	cellular response to ethylene stimulus
GO:0010260	0.043853	organ senescence
GO:0009723	0.045923	response to ethylene stimulus
GO:0051234	0.046034	establishment of localization
GO:0009581	0.046034	detection of external stimulus
GO:0070887	0.0463	cellular response to chemical stimulus
GO:0052545	0.0463	callose localization
GO:0009642	0.046495	response to light intensity
GO:0009695	0.046867	jasmonic acid biosynthetic process
GO:0033037	0.046867	polysaccharide localization
GO:0048518	0.046867	positive regulation of biological process
GO:0006970	0.046867	response to osmotic stress
GO:0010033	0.049274	response to organic substance
GO:0006811	0.049544	ion transport

Supplementary Table S5. Gene ontology enrichment analysis result for cellular component terms.

GO-ID	corr p-value	Description
GO:0005667	8.7661E-56	transcription factor complex
GO:0044451	4.4476E-50	nucleoplasm part
GO:0005654	8.8815E-50	nucleoplasm
GO:0031981	1.1701E-43	nuclear lumen
GO:0044428	6.4283E-41	nuclear part
GO:0070013	1.444E-40	intracellular organelle lumen
GO:0043233	1.444E-40	organelle lumen
GO:0031974	3.9833E-40	membrane-enclosed lumen
GO:0043234	1.3489E-26	protein complex
GO:0005634	3.9426E-23	nucleus
GO:0044446	5.2489E-20	intracellular organelle part
GO:0044422	5.2489E-20	organelle part
GO:0032991	2.9084E-19	macromolecular complex
GO:0043231	1.7052E-08	intracellular membrane-bounded organelle
GO:0043227	1.7052E-08	membrane-bounded organelle
GO:0043229	5.2527E-06	intracellular organelle
GO:0043226	5.2527E-06	organelle
GO:0044424	0.0013922	intracellular part
GO:0005622	0.0037627	intracellular
GO:0042729	0.018723	DASH complex
GO:0000780	0.038795	condensed nuclear chromosome, centromeric region
GO:0000778	0.038795	condensed nuclear chromosome kinetochore
GO:0000779	0.038795	condensed chromosome, centromeric region
GO:0000777	0.038795	condensed chromosome kinetochore

Supplementary Table S6. Gene ontology enrichment analysis result for molecular function terms.

GO-ID	P-value (Corrected)	Description
GO:0043565	1.0068E-74	sequence-specific DNA binding
GO:0003700	1.6862E-62	transcription factor activity
GO:0030528	7.3426E-60	transcription regulator activity
GO:0003677	9.7502E-42	DNA binding
GO:0003676	5.6189E-23	nucleic acid binding
GO:0005488	3.4153E-08	binding
GO:0005516	0.00010445	calmodulin binding
GO:0004012	0.0037518	phospholipid-translocating ATPase activity
GO:0005548	0.0052985	phospholipid transporter activity
GO:0005319	0.02205	lipid transporter activity

Supplementary Table S7. List of primers used for qPCR experiments

Gene ID	Forward primer	Reverse primer
Morus011258	5'-TTGGACAAATTGTTGTGCCT-3'	5'-GCCTCCCTCCACAACTGTAT-3'
Morus009921	5'-ATGCCAATGAACCAGAGACA-3'	5'-CACCTGAAATTCCAACATCG-3'
Morus009739	5'-CGTTTGCCACAGAAGAAGAA-3'	5'-CTTCTCCACCACCTTCCTTT-3'
Morus013971	5'-CAACTTCGATTGATGATGGG-3'	5'-TTCTGGAATTGTTTGGTTGC-3'
Morus002784	5'-CGTGCAATTCTCCTCTTGAA-3'	5'-AGAGACTACGGCCTCCTCAA-3'
Morus009509	5'-GCGGTTTAGTTCATCGGAAT-3'	5'-CGATCGTTGGTGTCAGTACC-3'
Morus013217	5'-GTTTAGCTCTTCGGCCTGTC-3'	5'-GGCGAAGTTCCTCTTTCTGA-3'
Morus012914	5'-AGAAATCGGAATTGAAACCG-3'	5'-TTAAACCCAACCTGGGAAAG-3'
Morus005757	5'-GCTGCTGATGAAGGAAGTGA-3'	5'-AGGAAATGGGCTCAAATGTC-3'
Actin	5'-GGCATCACTTAGCACCTTCCA-3'	5'-AATTGAAGGCCCGGACTCA-3'
